# Mitochondrial oxidants promote platelet activation and thrombotic susceptibility in prediabetes

**DOI:** 10.1172/JCI195662

**Published:** 2025-12-23

**Authors:** Azaj Ahmed, Pooja Yadav, Melissa Jensen, Katharine Geasland, Jagadish Swamy, Douglas R. Spitz, E. Dale Abel, Diana Jalal, Sanjana Dayal

**Affiliations:** 1 Department of Internal Medicine,; 2 Department of Radiation Oncology, and; 3 Holden Comprehensive Cancer Center, University of Iowa Carver College of Medicine, Iowa City, Iowa, USA.; 4 Department of Medicine, David Geffen School of Medicine, UCLA, Los Angeles, California, USA.; 5 Iowa City VA Healthcare System, Iowa City, Iowa, USA.

**Keywords:** Hematology, Vascular biology, Platelets

## Abstract

Recent studies suggest prediabetes is an independent risk factor for cardiovascular thrombotic events. However, the mechanisms that may promote platelet activation and thrombosis in prediabetes remain elusive. To determine mechanisms linking prediabetes and thrombosis as a function of age, we recruited military veterans with prediabetes and veterans who were normoglycemic, in young and middle-age groups. Compared with normoglycemic participants, platelets from those with prediabetes exhibited increased activation, mitochondrial oxidant load, mitochondrial membrane hyperpolarization, and greater thrombus formation ex vivo regardless of age. Preincubation of platelets with mitochondria-targeted antioxidants, such as SOD mimetic or mitoquinol (MitoQ), rescued this prothrombotic phenotype. These phenotypes were recapitulated in C57BL6/J mice exhibiting early onset of glucose intolerance when fed a high-fat (HF) diet for 2 weeks. Treatment of HF-fed mice with a SOD mimetic or MitoQ, or genetic overexpression of catalase within mitochondria, not only lowered mitochondrial oxidants, hyperpolarization, Ca^2+^ levels, and platelet activation but also protected against increased potential for carotid and pulmonary thrombosis. We also observed a bidirectional regulation of platelet activation by Ca^2+^ and mitochondrial oxidants. These findings support the idea that mitochondrial oxidant–dependent platelet activation induces a prothrombotic state in clinical prediabetes and preclinical models of short-term glucose intolerance and can be reversed by mitochondria-targeted antioxidants.

## Abstract 2

<strong>Our study demonstrates a link between prediabetes, platelet activation and thrombosis, and suggests a therapeutic potential for antioxidants that target mitochondrial oxidative stress. </strong>

## Graphical abstract

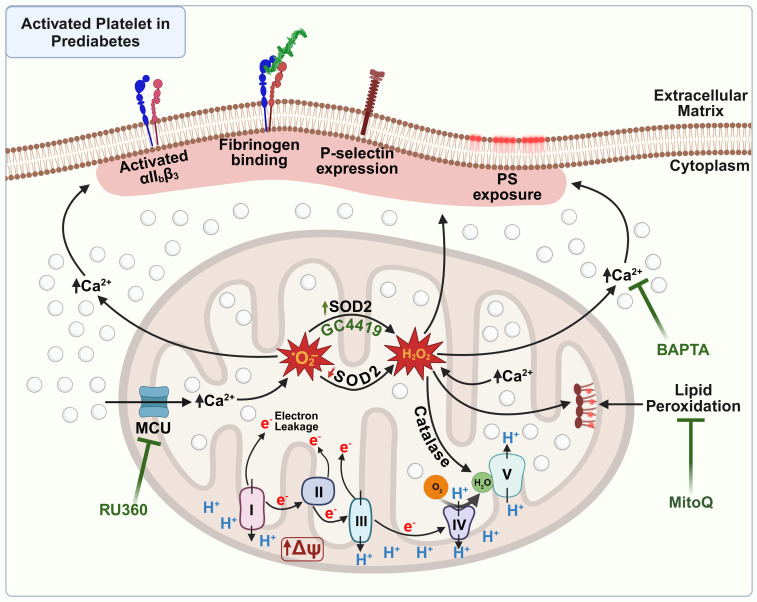

## Introduction

The prevalence of prediabetes, defined by the American Diabetes Association as a fasting blood glucose (FBG) level of 100–125 mg/dL and/or a hemoglobin A1c (HbA1c) of 5.7% to 6.4%, is increasing globally (1–3). Importantly, prediabetes is increasingly recognized as a risk factor not only for developing type 2 diabetes (T2D) but also for the development of cardiovascular diseases (CVDs). For example, in several large meta-analyses and prospective cohort studies, prediabetes was associated with increased risk of all-cause mortality and cardiovascular (CV) events (4–6). Furthermore, an umbrella assessment of systematic reviews and meta-analyses of prospective studies found that prediabetes raises risk for CVD by 39% compared with the risk for individuals with normal glucose levels (7). However, the mechanisms that underlie the association between prediabetes and thrombotic CVD are not known.

Emerging studies focusing on younger individuals suggest that this cohort is also vulnerable to prediabetes-associated CVD. In these individuals, prediabetes increases the risk of hospitalization for myocardial infarction (8) and is associated with worse prognosis after ischemic stroke (9). Interestingly, a study observed that though prediabetes was associated with greater risk for CVD and all-cause mortality in most age groups, the effect size was attenuated with increasing age (10). Hence, studying prediabetes in both young and older individuals has the potential to identify early cellular or metabolic abnormalities that may inform advocacy for additional preventive measures or for the development of therapeutic approaches.

Studies of middle-aged or elderly individuals or of aged mice from our laboratory and others have indicated that increased platelet activation that occurs with age is likely mediated by increase in platelet oxidants (11–14). Studies of animal models also suggest that approaches to lower platelet oxidants or hyperactivity prevent age-associated excessive thrombus formation (11, 15, 16). Using aged mice, we recently showed that this age-associated phenomenon is not regulated by Nox2-NADPH oxidase (17) but that mitochondria-generated oxidants (mito-oxidants) may be an important mediator (13). A similar phenomenon has been observed in patients with established T2D, in whom platelet mitochondrial dysfunction in association with modifications of mitochondrial antioxidant stress proteins was reported (18). Whether such a phenomenon also exists in young and aged individuals with prediabetes and mediates platelet hyperactivity and prothrombotic phenotype is not known.

Therefore, the objective of this study was 2-fold: first, to test that a prothrombotic platelet phenotype exists in younger and middle-aged adults with prediabetes and second, using ex vivo and in vivo approaches in clinical and preclinical models, respectively, to test whether the prothrombotic phenotype is mediated by the increased burden of intraplatelet mito-oxidants whose levels could be therapeutically targeted.

## Results

### Demographics, baseline characteristics, and complete blood cell count.

We studied 19 young (aged 18–50 years) and 19 middle-aged (51–64 years) military veterans with prediabetes. We also recruited 25 young and 22 middle-aged military veterans with normoglycemia (FBG < 100 mg/dL and HbA1c < 5.7%) as control participants. Demographics, baseline characteristics, serum chemistry findings, and complete blood cell counts of the patients and control participants are summarized in Table 1. The FBG, HbA1c, insulin, and Homeostasis Model Assessment of Insulin Resistance (HOMA-IR) values were increased in both young and middle-aged participants with prediabetes relative to normoglycemic young or middle-aged control participants, respectively. Other parameters, such as systolic BP (SBP), diastolic BP (DBP), cholesterol, LDL, HDL, and triglyceride levels, were mostly not different among groups, but an increase in SBP in middle-aged participants with prediabetes or DBP in middle-aged control participants and a decreased HDL level in young participants with prediabetes were noted. The complete blood cell counts also were comparable among the groups except that an increase in neutrophil count was noted in young participants with prediabetes and an increase in the neutrophil/lymphocyte ratio was observed in middle-aged control participants. One and 2 patients in young control and young prediabetic groups, respectively, were taking antihypertensive drugs. In the middle-aged control group, 3 individuals were taking lipid-lowering drugs, and 1 was taking antihypertensive medication. In the middle-aged prediabetic group, 3 participants were taking lipid-lowering drugs, and 4 were taking antihypertensive medication.

### Prediabetes enhanced agonist-induced platelet activation and thrombus formation ex vivo.

**Figure 1:**
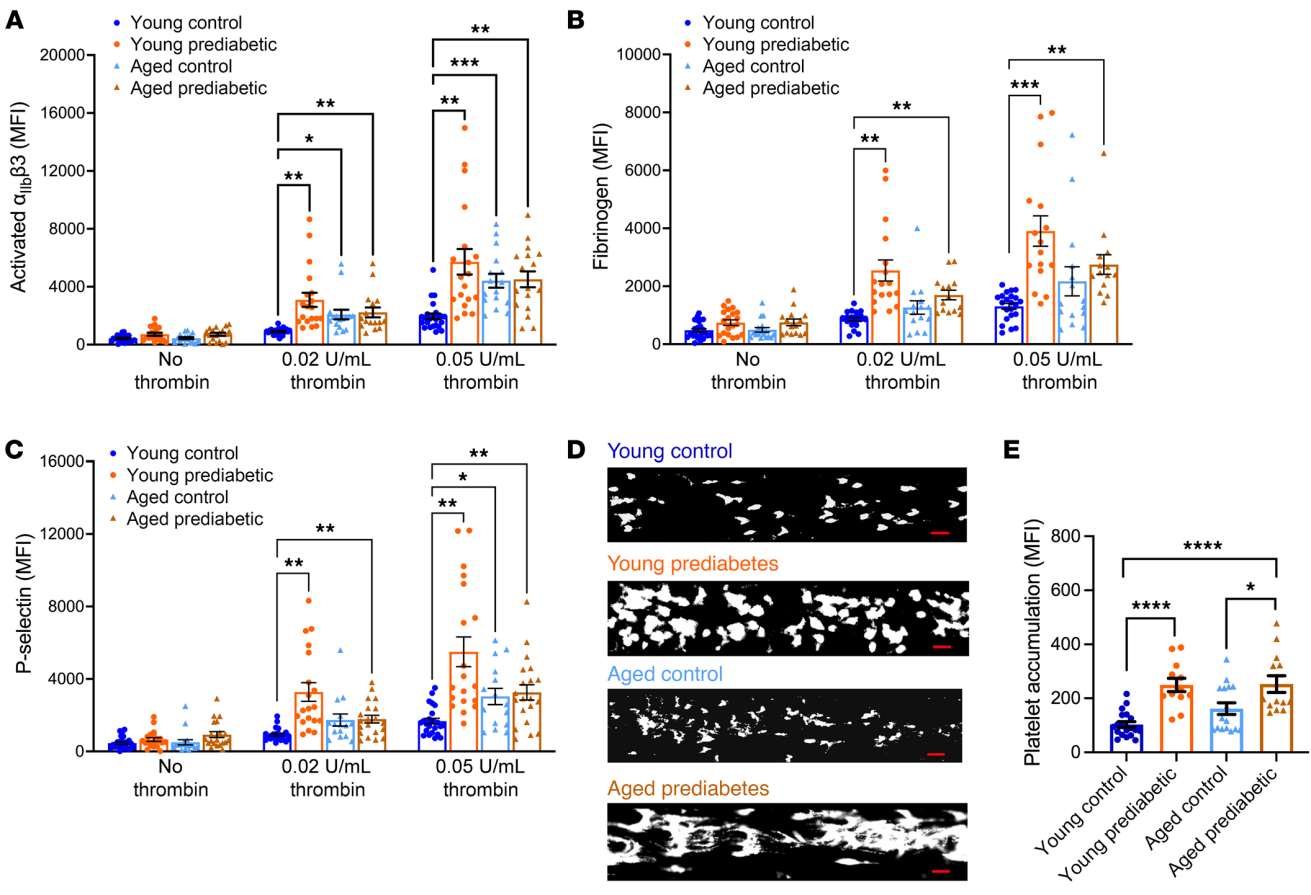
Platelets from patients with prediabetes show hyperactivity at both young and older ages. Washed platelets were incubated with antibody against (**A**) activated α_IIb_β3, (**B**) fibrinogen, or (**C**) P-selectin, in the absence or presence of thrombin (*n* = 2 doses) for 10 minutes; diluted in PBS; fixed; and analyzed via flow cytometry. Data are presented as mean ± SEM and analyzed using 2-way ANOVA with Tukey’s test for multiple-group comparisons on log-transformed values. *n* = 14–24/group. (**D**) Calcein green–labeled washed platelets were suspended in Tyrode’s buffer and superfused over a collagen-coated microfluidic chamber under arterial shear stress. Representative images of platelet accumulation after 5 minutes of superfusion for each group are shown. Scale bar: 100 μm. (**E**) Quantification of **D** is shown as MFI, presented as mean ± SEM and analyzed using 2-way ANOVA with Tukey’s test for multiple-group comparisons on log-transformed values. *n* = 12–19/group. *P < 0.05, **P < 0.01, ***P < 0.001, ****P < 0.0001.

To evaluate alterations in platelet activation as a function of prediabetes or age, we examined markers for integrin activation (i.e., activated α_IIb_β3), fibrinogen binding, and granule release (P-selectin) in the washed platelets in the absence (resting state) or presence (activated state) of the agonist, thrombin. There were no alterations for these markers at resting state among 4 groups (Figure 1, A–C). Two submaximal doses of thrombin (0.02 and 0.05 U/mL) were used to capture differential sensitivity of platelets due to prediabetes or age, and we observed a dose-dependent increase in mean fluorescence for these platelet activation markers in all 4 groups.

After activation, the main effect of prediabetes was evident for all 3 activation markers within each dose of thrombin. A significant increase due to age among normoglycemic participants was observed for activated α_IIb_β3 at both doses of thrombin and for P-selectin at the higher dose of thrombin. However, among the participants with prediabetes, increased platelet activation with advancing age was not evident. In fact, young participants with prediabetes had an increasing trend for platelet activation compared with the middle-aged participants with prediabetes. Middle-aged participants with prediabetes did not have any significant increase when compared with the middle-aged normoglycemic participants for any of the platelet activation markers.

Platelet accumulation and ex vivo thrombus formation were assessed in a microfluidic flow chamber after 5 minutes of superfusion on the collagen coated surface. Prediabetes was associated with significant increase in ex vivo thrombus formation in both young and middle-aged participants (Figure 1, D and E). There was also a greater accumulation of platelets in middle-aged participants with prediabetes compared with the middle-aged normoglycemic group.

### Prediabetes aggravated platelet mito-oxidant levels, aggravated mitochondrial membrane hyperpolarization, and increased phosphatidylserine exposure.

**Figure 2:**
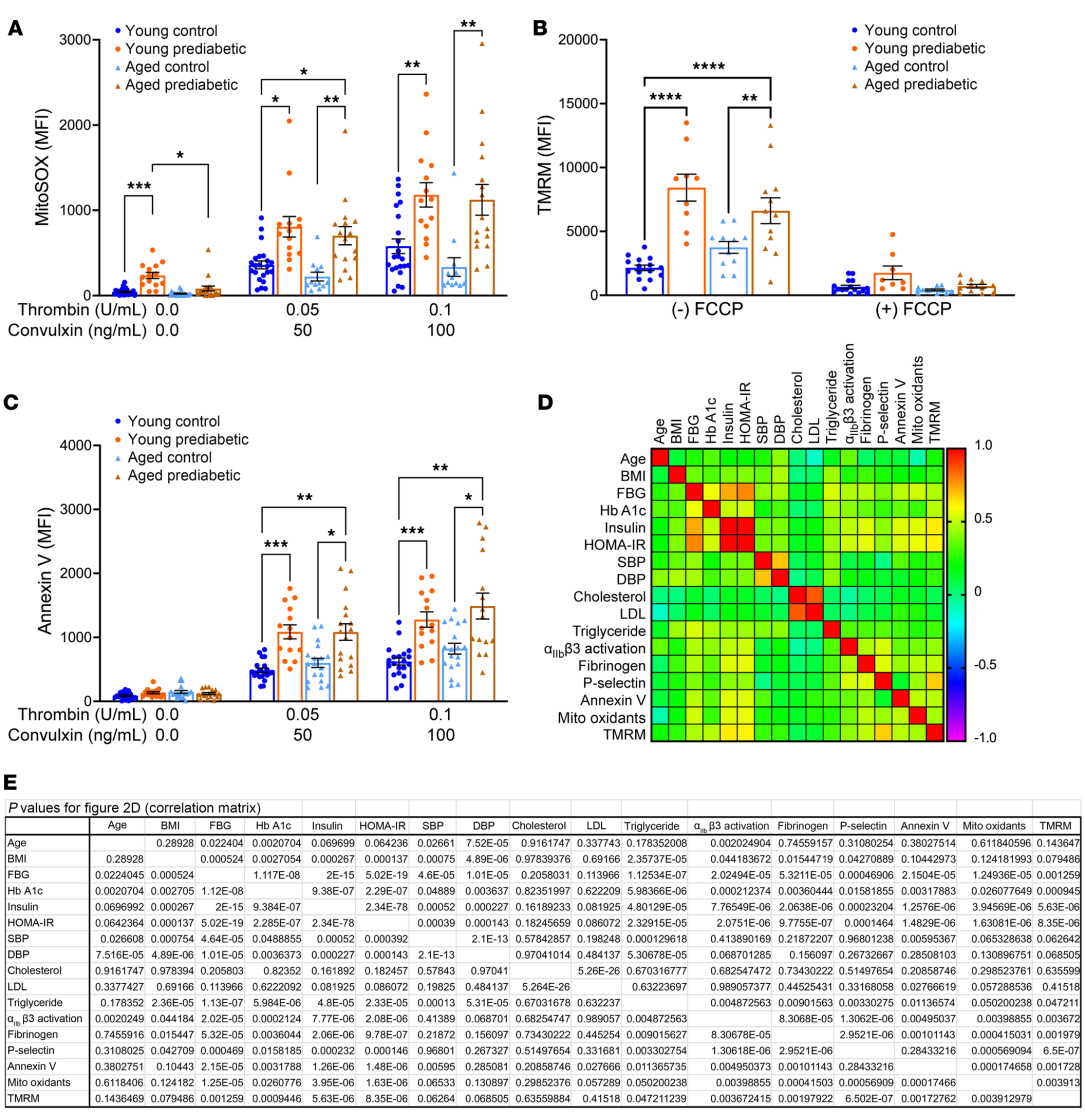
Platelets from participants with prediabetes have increased concentrations of mito-oxidants, mitochondrial membrane hyperpolarization, and PS exposure. Washed platelets were prepared, and (**A**) mitochondrial pro-oxidants were detected by incubating platelets with 2.5 μM MitoSOX in the absence or presence of thrombin and convulxin; (**B**) Δψm was detected by incubating platelets with TMRM dye for 10 minutes in the presence or absence of FCCP; and (**C**) PS exposure was measured by annexin V binding in washed platelets in the absence or presence of thrombin and convulxin activation. Samples were evaluated via flow cytometry, and MFI data are presented as mean ± SEM and analyzed using 2-way ANOVA with Tukey’s test for multiple-group comparisons within each treatment. *n* = 9–22/group. *P < 0.05, **P < 0.01, ***P < 0.001, ****P < 0.0001. (**D**) A heatmap showing associations among different variables using the Spearman’s correlation. *n* = 85. (**E**) Correspondent *P* values for **D**.

We next tested whether prediabetes or age was associated with increased platelet mito-oxidants in our cohort. Using the oxidative fluorescent dye MitoSOX, we measured accumulation of oxidants within mitochondria. Under resting conditions, increased MitoSOX fluorescence was observed in the young participants with prediabetes compared with the young control participants and middle-aged participants with prediabetes (Figure 2A). Upon agonist activation, an increase in MitoSOX fluorescence was seen in all groups, but enhancement in the participants with prediabetes exceeded the increase in age-matched control groups. The measurement of mitochondrial membrane potential (Δψm) using tetramethylrhodamine, methyl ester (TMRM), fluorescence revealed mitochondrial hyperpolarization in both groups with prediabetes compared with age-matched control groups (Figure 2B). Treatment with the uncoupler carbonyl cyanide-*p*-trifluoromethoxyphenylhydrazone (FCCP) collapsed the Δψm in all groups; the residual Δψm remained modestly higher in those with prediabetes; but the difference from the control group was not significant. Annexin V binding demonstrating phosphatidylserine (PS) exposure in platelets was similar in the resting state among the 4 groups but was increased upon agonist stimulation in platelets from both the young and middle-aged participants with prediabetes relative to the age-matched control participants (Figure 2C). In contrast with findings for integrin activation, fibrinogen binding, or P-selectin, the middle-aged participants with prediabetes had a significant increase in MitoSOX and TMRM fluorescence and PS exposure compared with the middle-aged normoglycemic participants, suggesting an additive effect of prediabetes in middle-aged individuals for these parameters.

### Correlation matrix.

To assess the relationships among age, FBG, HbA1c, insulin, HOMA-IR, BMI, BP, lipids, and platelet activation parameters, a correlation matrix analysis was performed (Figure 2, D and E). Age was associated positively with FBG, HbA1c, SBP (*P* < 0.05 to *P* < 0.01), and DBP (*P* < 0.000001) and with only 1 marker of platelet activation (i.e., activated integrin, *P* < 0.01). Likewise, BMI was associated positively with FBG, HbA1c, insulin, HOMA-IR, SBP, DBP, and triglyceride values (*P* < 0.05 to *P* < 0.000001), and with platelet integrin activation, as well as with fibrinogen and P-selectin (*P* < 0.05 for all 3 markers). There was no association of age with BMI in our cohort. All the markers of platelet activation showed the strongest associations with HOMA-IR, insulin, and FBG (*P* < 0.001 to *P* < 0.0000001), and HbA1c (*P* < 0.05 to *P* < 0.0001). There were no associations between platelet activation markers and DBP or total cholesterol. LDL and SBP were associated with only annexin V (*P* < 0.05 and *P* < 0.01, respectively), and triglycerides had significant associations with most platelet activation markers (*P* < 0.05 to *P* < 0.01). Both mito-oxidant and TMRM staining were positively associated with all the platelet activation markers (*P* < 0.01 to *P* < 0.0001), suggesting a mechanistic link.

### A SOD2 mimetic (GC4419) and lipid peroxide–targeting drug, mitoquinol, lowered mito-oxidant burden and decreased platelet activation and ex vivo thrombus growth in prediabetes.

Given the elevated burden of platelet mito-oxidants in prediabetes, we questioned whether this increase is due to a compromised expression or activity of the SOD2 enzyme, which is exclusively expressed in mitochondria where it dismutates superoxide. No alteration in levels of SOD2 protein by Western blot as a function of age or prediabetes was observed (Supplemental Figure 1, A and B; supplemental material available online with this article; https://doi.org/10.1172/JCI195662DS1). Considering that posttranslational modifications may contribute to impaired mitochondrial antioxidant defense despite unaltered protein expression, we measured SOD2 activity and observed significant reduction in the participants with prediabetes (Supplemental Figure 1C).

**Figure 3:**
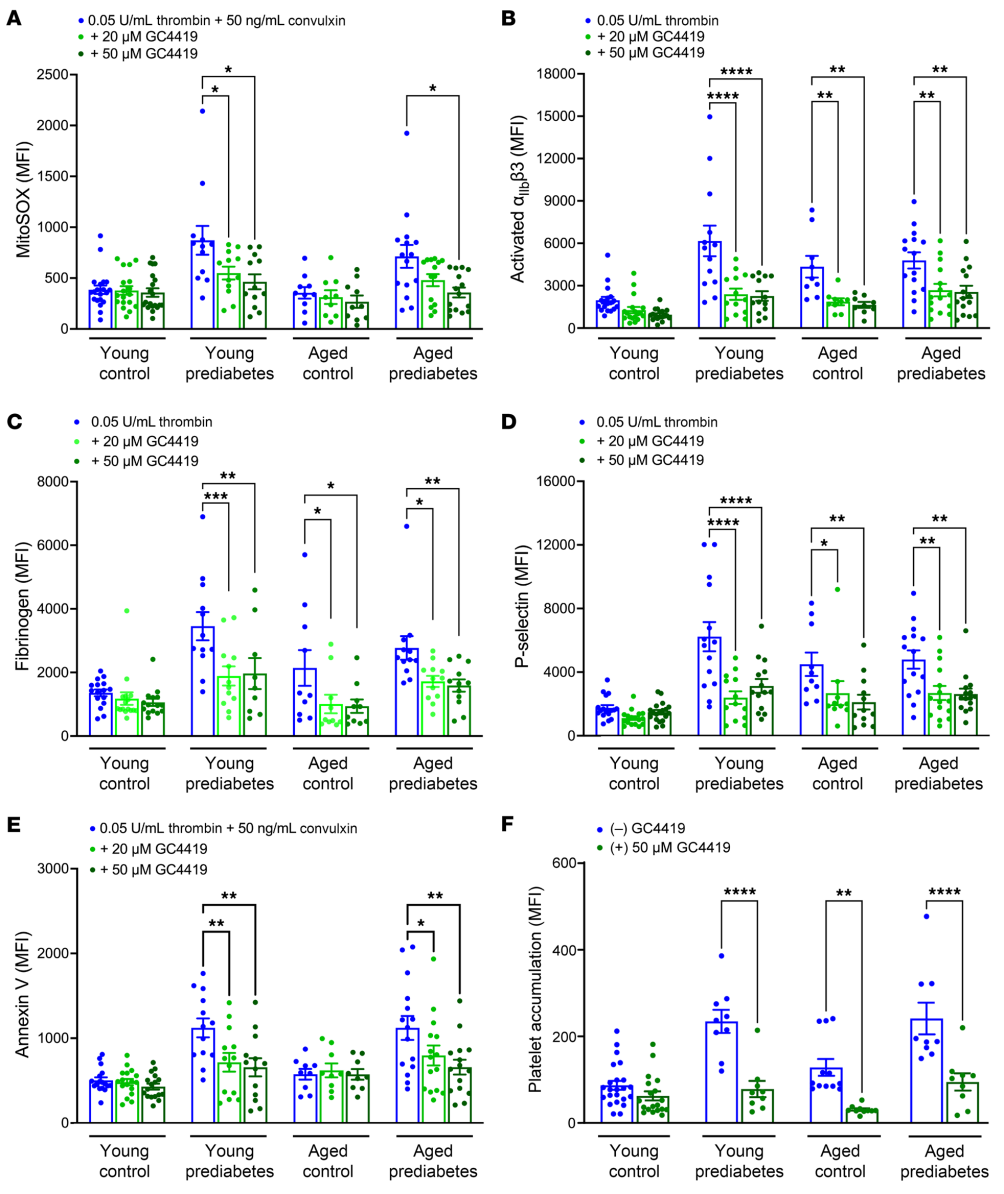
Treatment of platelets with SOD mimetic (GC4419) lowers mito-oxidant burden and decreases agonist-induced platelet activation and ex vivo thrombus formation in prediabetes. Washed platelets were preincubated either with vehicle buffer (control) or with 25 or 50 μM GC4419 for 10 minutes, then stained with either (**A**) MitoSOX dye or fluorescent antibody against (**B**) activated α_IIb_β3, (**C**) fibrinogen, (**D**) P-selectin, or with (**E**) annexin V; activated with thrombin or thrombin plus convulxin for 10 minutes; and analyzed via flow cytometry. Data are presented as mean ± SEM and analyzed using 2-way ANOVA with Tukey’s analysis for multiple-group comparisons, whereby, within each cohort, platelets treated with 2 doses of GC4419 were compared with the control treatment. *n* = 9–20/group. (**F**) Calcein green–labeled washed platelets were preincubated with 50 μM GC4419 and superfused over a collagen-coated microfluidic chamber under arterial shear stress for 5 minutes. MFI was quantified, presented as mean ± SEM, and analyzed using Student’s *t* test within each cohort. *n* = 9–19/group. *P < 0.05, **P < 0.01, ***P < 0.001, ****P < 0.0001.

We then tested whether supplementing platelets with a small-molecule SOD mimetic, avasopasem (GC4419; Galera Therapeutics) (13), could decrease mito-oxidants, platelet activation, and the likelihood of ex vivo platelet thrombus formation. GC4419 is taken up by mitochondria and mimics the activity of SOD2 (19). Ex vivo treatment with 2 doses of GC4419 protected platelets from prediabetes-induced hyperactivity in both young and middle-aged groups, manifested by a significant lowering of the mito-oxidant load as well as reduced integrin activation, fibrinogen binding, granule release, and PS exposure (Figure 3, A–E). GC4419 also lowered integrin activation, fibrinogen, and granule release in the middle-aged normoglycemic participants. Furthermore, GC4419 prevented excessive platelet thrombus formation on collagen under shear stress conditions in both prediabetes groups and in the middle-aged normoglycemic participants (Figure 3F and Supplemental Figure 2A).

**Figure 4:**
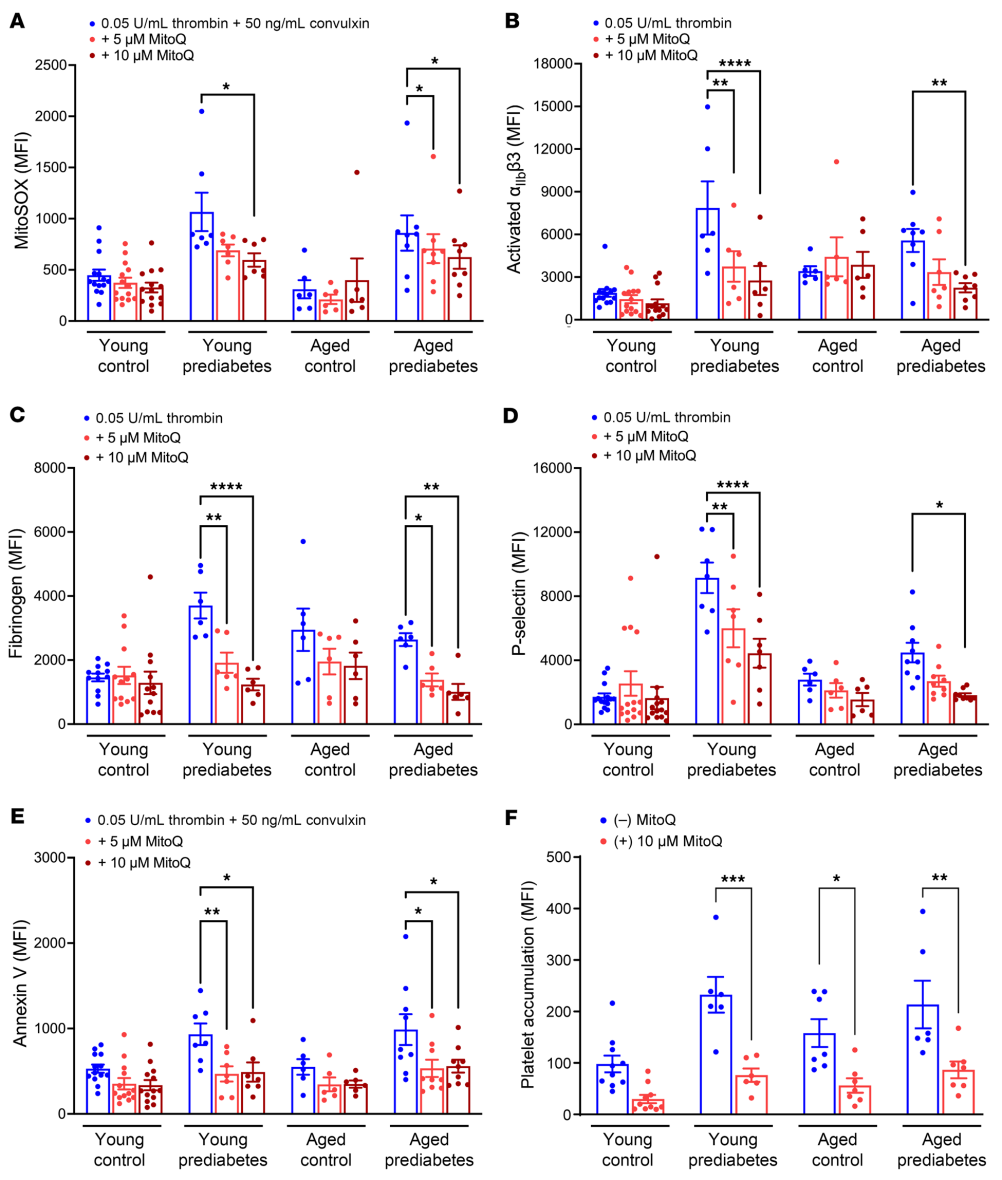
Treatment of platelets with MitoQ lowers mito-oxidant burden and decreases agonist-induced platelet activation and ex vivo thrombus formation in prediabetes. Washed platelets were preincubated either with vehicle buffer (control) or with 5 or 10 μM MitoQ for 15 minutes, then stained with (**A**) MitoSOX dye or fluorescent antibody against (**B**) activated α_IIb_β3, (**C**) fibrinogen, (**D**) P-selectin, or (**E**) annexin V, following activation with thrombin or thrombin plus convulxin for 10 minutes, and analyzed via flow cytometry. Data are presented as mean ± SEM and analyzed using 2-way ANOVA with Tukey’s analysis for multiple-group comparisons, whereby, within each cohort, platelets treated with 2 doses of MitoQ were compared with the control treatment. *n* = 6–14/group. (**F**) Calcein green–labeled washed platelets were preincubated with 10 μM MitoQ and superfused over a collagen-coated microfluidic chamber under arterial shear stress for 5 minutes. MFI was quantified, presented as mean ± SEM, and analyzed using Student’s *t* test within each cohort. *n* = 6–10/group. *P < 0.05, **P < 0.01, ***P < 0.001, ****P < 0.0001.

As a complementary approach, we treated platelets with the mitochondria-targeted antioxidant mitoquinol (MitoQ), which is positively charged with a triphenyl phosphonium ion and accumulates in mitochondria, where it decreases lipid peroxidation by acting as a chain-breaking antioxidant by neutralizing lipid peroxyl radicals (20). Prior treatment of platelets with MitoQ prevented enhancement of mito-oxidants, integrin activation, fibrinogen binding, granule release, and PS exposure in both prediabetes groups but not in any control groups (Figure 4, A–E). Treatment with MitoQ also protected from excessive platelet accumulation on collagen under shear stress in the young age group with prediabetes and both middle-aged normoglycemic and middle-aged prediabetes groups (Figure 4F and Supplemental Figure 2B).

### Mice with early onset of glucose intolerance developed a prothrombotic phenotype that is preventable with a SOD mimetic.

To test the antiplatelet and antithrombotic efficacy of the SOD mimetic (GC4419) in an in vivo system, we established a mouse model mimicking early-onset glucose intolerance, which we used as a model of prediabetes. C57BL6/J mice were fed a high-fat (HF) diet for a short duration (2 weeks). Relative to chow-fed mice, HF-fed mice gained about 10% more weight and exhibited glucose intolerance, increased fasting and random glucose levels, and higher insulin level at baseline and 30 minutes after dextrose injection (1 g/kg) (Supplemental Figure 3, A–E). Platelet flow cytometry revealed that the mice fed the HF diet for 2 weeks had increased platelet integrin activation, granule release, PS exposure, and mito-oxidant levels compared with chow-fed mice (Supplemental Figure 3, F–I). These findings mimic the findings in the human participants with prediabetes.

To test the protective effects of GC4419, 1 week after mice were fed the HF or chow diet, GC4419 (10 mg/kg daily via i.p. injection) or vehicle buffer was administered (13), and platelets were harvested after 1 week of treatment. Treatment with GC4419 did not affect agonist-induced platelet activation or mito-oxidant levels in mice fed the chow diet; however, it prevented the increase in mito-oxidants, integrin activation, P-selectin expression, and PS exposure in mice fed the HF diet (Supplemental Figure 4, A–D). Importantly, although the photochemical injury to the carotid artery caused a significantly shorter time to form an occlusive thrombus in HF-fed mice treated with vehicle buffer relative to mice fed the chow diet, treatment with GC4419 prevented HF-fed mice from this accelerated thrombus formation (Supplemental Figure 4E).

In a complementary model of platelet activation-induced pulmonary thrombosis, infusion of collagen in mice treated with vehicle buffer caused rapid death in HF-fed mice compared with chow-fed mice. Treatment with GC4419 prolonged time to death in HF-fed mice to rates similar to those of chow-fed mice treated with buffer or GC4419 (Supplemental Figure 4F). Bleeding time assessed by tail transection was similar in all groups regardless of diet or treatment with GC4419 (Supplemental Figure 4G). Additionally, treatment with GC4419 did not alter glucose tolerance test (GTT) results in mice fed the chow or HF diet (Supplemental Figure 5, A and B).

### Treatment of mice fed the HF diet with MitoQ reduced mito-oxidant levels, platelet activation, and in vivo thrombosis in part by influencing platelet Ca^2+^ mobilization.

**Figure 5:**
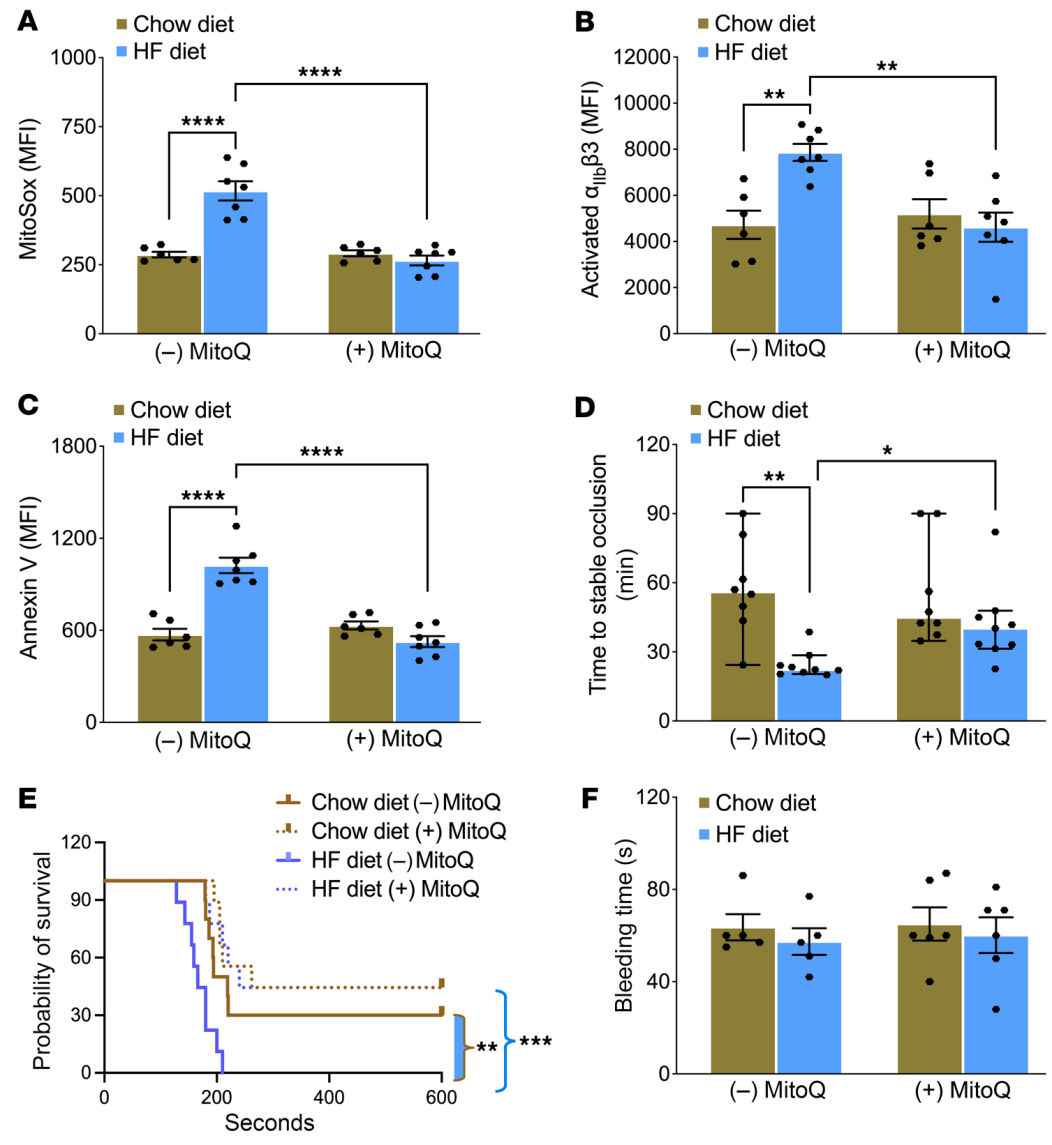
In vivo treatment with MitoQ protects mice with short-term glucose intolerance from enhanced platelet mito-oxidants, platelet activation, and susceptibility to carotid artery and pulmonary thrombosis without altering hemostasis. C57BL6/J mice fed chow or an HF diet for 2 weeks received daily treatment with (+) MitoQ (1 mg/kg daily, i.p.) or vehicle buffer (–) MitoQ beginning after 1 week on the diet. Washed platelets were prepared for quantifying (**A**) mito-oxidants, (**B**) α_IIb_β3 activation, and (**C**) annexin V binding; activated with 0.05 U/mL thrombin and 50 ng/mL convulxin (**A** and **C**) and with 0.05 U/mL thrombin for **B**; and analyzed via flow cytometry. (**D**) Time to stable occlusion of the carotid artery after photochemical injury. Data are presented as median with 95% CI and analyzed by Kruskal-Wallis test with Dunn’s post hoc test for multiple-group comparisons (*n* = 8–9 per group). (**E**) Survival curve depicting time to death after infusion with 0.5 μg/g collagen. Data were analyzed using the Mantel-Cox log-rank test. The comparison between HF groups [(–) or (+) MitoQ] is shown with a blue bracket and between chow and HF-fed (–) MitoQ groups is shown as a brown bracket with blue fill (*n* = 9–10 per group). (**F**) Tail bleeding time. Data for **A**–**C** and **F** are presented as mean ± SEM and analyzed using 2-way ANOVA with Tukey’s test for multiple-group comparisons (*n* = 5–7 per group). *P < 0.05, **P < 0.01, ***P < 0.001, ****P < 0.0001.

To test the protective effects of MitoQ, we treated mice with MitoQ (1 mg/kg daily via i.p. injection) or vehicle buffer starting a week after they were fed the HF or chow diet. After a week of MitoQ treatment, platelets were isolated from whole blood. Although in vivo treatment with MitoQ did not affect agonist-induced platelet mito-oxidant generation or platelet activation in mice fed the chow diet, it prevented mice fed the HF diet from increased mito-oxidants, integrin activation, and PS exposure (Figure 5, A–C). In HF-fed mice treated with vehicle buffer, time to stable occlusion of the carotid artery after photochemical injury was significantly shorter relative to mice fed the chow diet, and treatment with MitoQ prevented this accelerated thrombosis in HF-fed mice (Figure 5D). Furthermore, the mice fed HF diet treated with vehicle buffer died rapidly with infusion of collagen compared with chow-fed mice, and treatment with MitoQ prolonged time to death in HF-fed mice similar to chow-fed mice treated with buffer or MitoQ (Figure 5E). Bleeding time assessed by tail transection was similar in all groups regardless of diet or treatment with MitoQ (Figure 5F). Treatment with MitoQ also did not alter GTT results in mice fed the chow or HF diet (Supplemental Figure 6, A and B).

**Figure 6:**
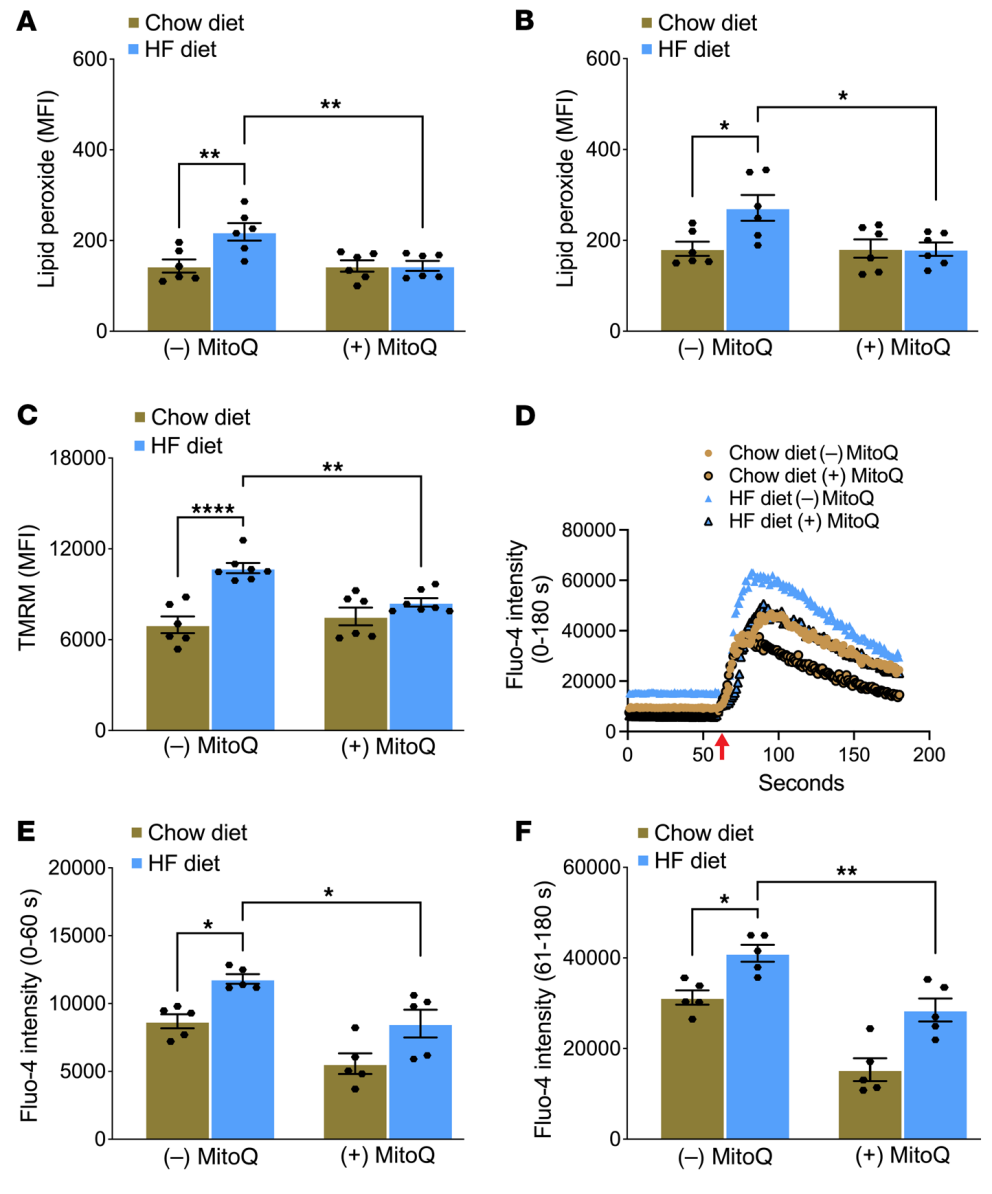
In vivo treatment with MitoQ protects mice with short-term glucose intolerance from enhanced generation of platelet lipid peroxides, mitochondrial membrane hyperpolarization, and increased platelet Ca^2+^. C57BL6/J mice fed chow or an HF diet for 2 weeks received daily treatment with (+) MitoQ (1 mg/kg daily, i.p.) or vehicle buffer [(–) MitoQ] beginning after 1 week on the diet. Washed platelets were prepared 1 week after treatment with MitoQ, and lipid peroxides were quantified using flow cytometry after activation with (**A**) 0.05 U/mL thrombin or (**B**) 0.05 U/mL thrombin and 50 ng/mL convulxin. (**C**) TMRM fluorescence was measured in washed platelets. (**D**) Representative tracings of platelet Ca^2+^ content measured with Fluo-4 dye at baseline for up to 60 seconds and then with 0.05 U/mL thrombin for another 120 seconds. The addition of thrombin is indicated by the red arrow. Quantification for **D** is shown (**E**) at baseline and (**F**) after thrombin activation. Data for **A**–**C**, **E**, and **F** are presented as mean ± SEM and analyzed using 2-way ANOVA with Tukey’s test for multiple-group comparisons (*n* = 5–7 per group). *P < 0.05, **P < 0.01, ****P < 0.0001.

To gain mechanistic insight into the role of MitoQ, we first measured lipid peroxide levels and Δψm in platelets. In vehicle buffer–treated mice, we observed that platelets from mice fed the HF diet, compared with mice fed the chow diet, had increased lipid peroxide levels when activated with thrombin or thrombin plus convulxin (Figure 6, A and B) and a higher Δψm (Figure 6C). Treatment of mice with MitoQ normalized the differences observed between chow diet– or HF diet–fed mice for both parameters. Furthermore, in the absence of MitoQ, mice fed the HF diet not only had increased baseline total cellular Ca^2+^ levels but also had increased levels with thrombin. Treatment with MitoQ normalized the differences in Ca^2+^ levels between chow diet– or HF diet–fed mice at baseline as well as in the thrombin-triggered increase (Figure 6, D–F).

At this point, we considered that our interpretation of elevated mito-oxidant levels based on increased MitoSOX fluorescence in platelets from HF-fed mice could be confounded by the simultaneous presence of increased TMRM fluorescence, because accumulation of both dyes could occur in hyperpolarized mitochondria. To address this possibility, we stained the platelets from chow diet– and HF diet–fed mice with TMRM and MitoSOX, where we first confirmed an increased signal for both TMRM and MitoSOX in platelets from mice fed the HF diet compared with chow-fed mice (Supplemental Figure 7, A and B). Addition of FCCP reduced the TMRM signal profoundly in all mice regardless of diet but only caused a nonsignificant drop in MitoSOX fluorescence. Additionally, a higher MitoSOX signal was maintained in the platelets from mice fed the HF diet, even after FCCP treatment, but the signal was normalized after treatment with a SOD mimetic or MitoQ. Together, these data suggest MitoSOX accumulation in our model is minimally driven by Δψm, and the increased accumulation of MitoSOX in platelets from HF diet–fed mice under the study conditions reflects elevation in mito-oxidants.

To determine the contribution of altered calcium mobilization in the platelet phenotype in mice fed the HF diet, we used the intracellular calcium chelator BAPTA-AM and the mitochondrial calcium uniporter complex inhibitor Ru360, which specifically inhibits calcium influx into mitochondria. The increase in intracellular calcium in platelets from mice fed the HF diet at baseline or with thrombin activation was attenuated by the BAPTA-AM but remained unaffected by the Ru360. This suggests that, under these conditions, the driver of increased platelet Ca^2+^ is via mechanisms related to platelet plasma membrane Ca^2+^ uptake and/or its release intracellularly but not by increased mitochondrial Ca^2+^ entry (Supplemental Figure 8, A and B). However, Ru360 exposure completely depolarized mitochondria (Supplemental Figure 8C). BAPTA-AM also decreased Δψm but had less of an effect than Ru360, suggesting a greater role of mitochondrial Ca²^+^ in sustaining the increase in Δψm. Exposure to either BAPTA-AM or Ru360 reduced mito-oxidants, including lipid peroxides and integrin activation in platelets from HF-fed mice, supporting Ca^2+^ dependency of these pathophysiologic changes (Supplemental Figure 8, D–F).

### Overexpression of mitochondria-targeted catalase protects glucose-intolerant mice from platelet activation and thrombosis.

**Figure 7:**
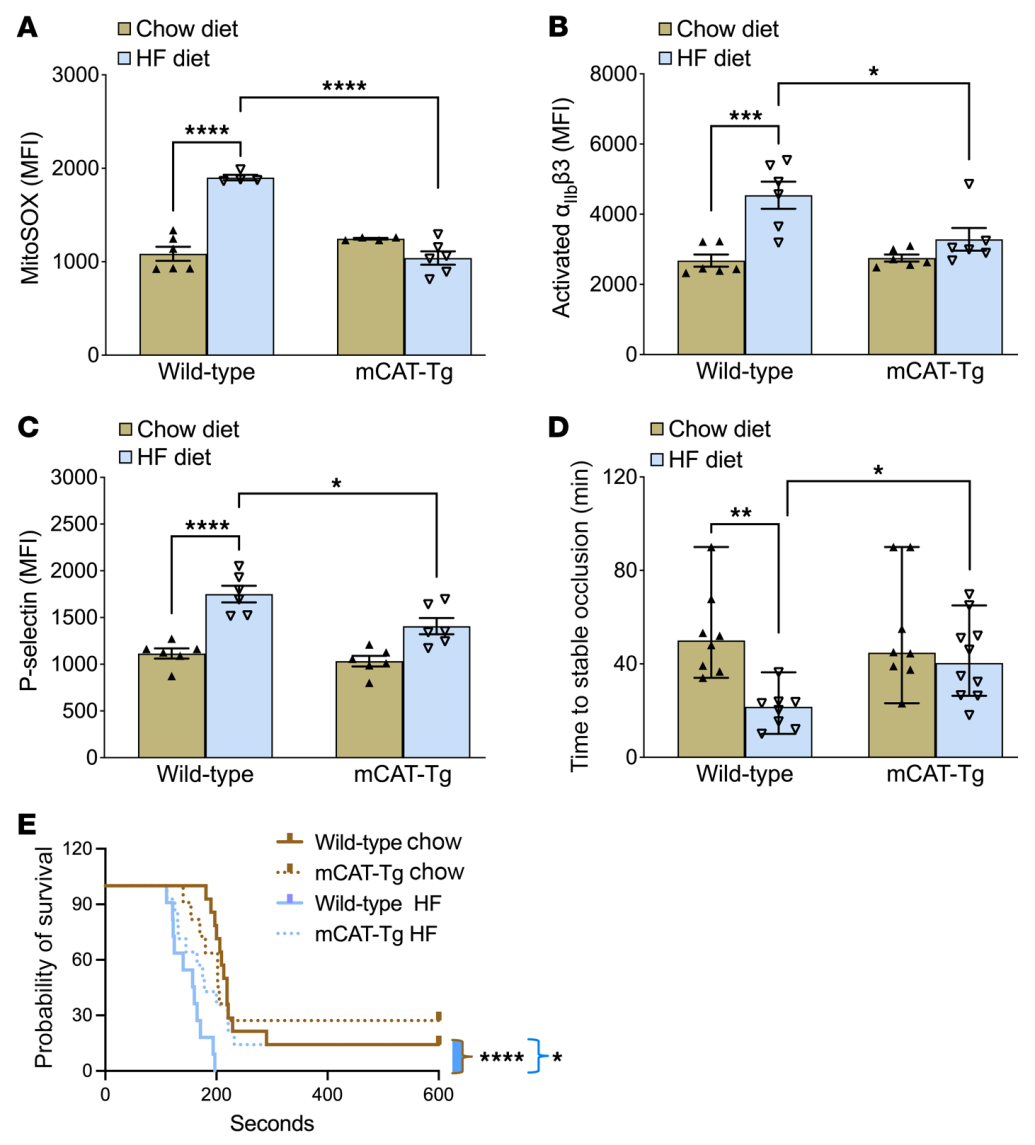
mCAT-Tg mice are protected from enhanced platelet mito-oxidants, platelet activation, and susceptibility to carotid artery and pulmonary thrombosis after short-term HF diet feeding. mCAT-Tg mice and wild-type littermates were fed chow or an HF diet for 2 weeks. Washed platelets were prepared for quantifying (**A**) mito-oxidants, (**B**) α_IIb_β3 activation, and (**C**) P-selectin expression after activation with 0.05 U/mL thrombin and 50 ng/mL convulxin for **A**, and with 0.05 U/mL thrombin for **B** and **C**, and analyzed via flow cytometry. (**D**) Time to stable occlusion of the carotid artery after photochemical injury. Data are presented as median with 95% CI and were analyzed by Kruskal-Wallis test with Dunn’s post hoc test for multiple-group comparisons (*n* = 8–10 per group). (**E**) Survival curve depicting time to death after infusion with 0.5 μg/g collagen. Data were analyzed using the Mantel-Cox log-rank test. The comparison between wild-type and mCAT-Tg mice fed the HF diet is shown with a blue bracket and between chow- and HF-fed wild-type groups is shown as a brown bracket with blue fill (*n* = 11–14 per group). Data for **A**–**C** are presented as mean ± SEM and analyzed using 2-way ANOVA with Tukey’s test for multiple-group comparisons (*n* = 6 per group). *P < 0.05, **P < 0.01, ***P < 0.001, ****P < 0.0001.

Finally, we used a genetic approach to test whether reducing mitochondrial peroxide protects mice from platelet activation and thrombosis. We fed the HF or chow diet for 2 weeks to mCAT-Tg mice (mice overexpressing catalase in mitochondria) and their wild-type littermate controls. Wild-type and mCAT-Tg littermates fed the HF diet developed glucose intolerance (Supplemental Figure 9, A and B). Wild-type mice fed the HF diet for 2 weeks had increased platelet mito-oxidants, integrin activation, and granule release compared with mice fed the chow diet (Figure 7, A–C). In contrast, the mCAT-Tg mice fed HF diet had decreased platelet mito-oxidants and activation relative to wild-type mice fed the HF diet.

**Figure 8:**
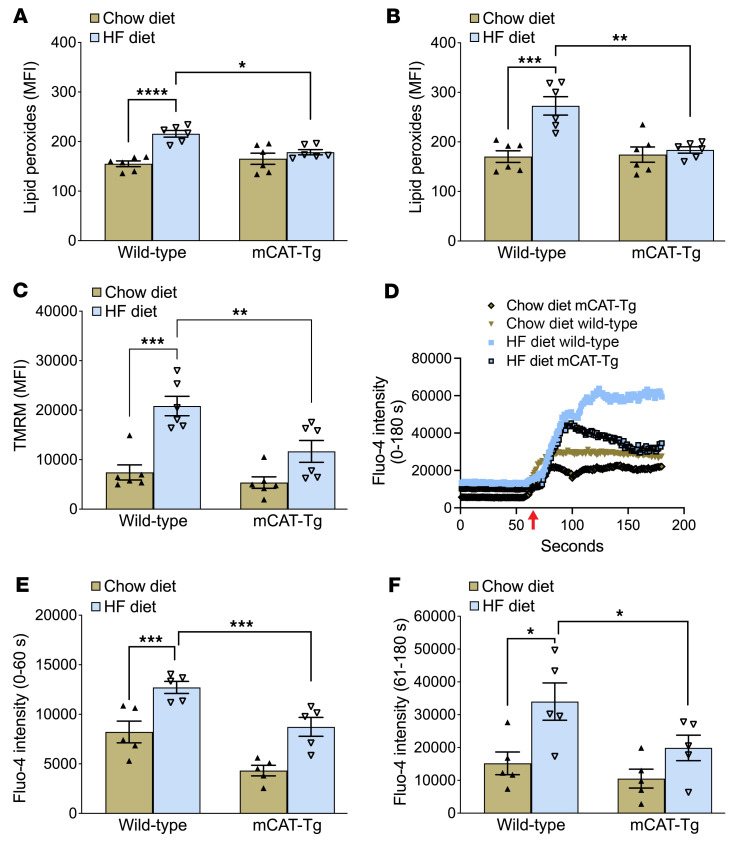
mCAT-Tg mice are protected from excessive accumulation of platelet lipid peroxides, mitochondrial membrane hyperpolarization, and increased platelet Ca^2+^ after short-term HF diet feeding. Washed platelets were prepared 2 weeks after mice were fed chow or an HF diet, and lipid peroxides were quantified after activation with (**A**) 0.05 U/mL thrombin or (**B**) 0.05 U/mL thrombin and 50 ng/mL convulxin, then analyzed via flow cytometry. (**C**) TMRM fluorescence was measured in washed platelets. (**D**) Representative tracings of Ca^2+^ flux measured with Fluo-4 dye at baseline for up to 60 seconds and after 0.05 U/mL thrombin for another 120 seconds. The red arrow indicates the addition of thrombin. Quantification for **D** is shown (**E**) at baseline and (**F**) with thrombin activation. Data for **A**–**C**, **E**, and **F** are presented as mean ± SEM and analyzed using 2-way ANOVA with Tukey’s test for multiple-group comparisons (*n* = 5–7 per group). *P < 0.05, **P < 0.01, ***P < 0.001, ****P < 0.0001.

Furthermore, in wild-type mice, the HF diet shortened the time to form stable occlusion compared with the time to occlusion in mice fed the chow diet or mCAT-Tg mice fed the HF diet (Figure 7D). In the pulmonary thrombosis model, the HF diet caused rapid death in wild-type mice, whereas mCAT-Tg littermates had a prolonged survival (Figure 7E). Mechanistically, the HF diet enhanced lipid peroxides, Δψm, and baseline Ca^2+^, as well as Ca^2+^ elevation with thrombin in wild-type mice but not in the mCAT-Tg mice (Figure 8, A–F).

Together, these findings indicate mitochondrial hyperpolarization and elevation in platelet Ca^2+^ and mito-oxidants, including lipid peroxide, are linked to platelet activation.

## Discussion

Despite the significant associations reported between prediabetes and thrombotic CV outcomes (4–6), the mechanisms driving the prothrombotic state in prediabetes are incompletely understood. Using pharmacological and genetic approaches in a clinical and preclinical model of prediabetes, we report several important findings. First, we demonstrate a link between human prediabetes and markers of platelet activation, mito-oxidant burden, and ex vivo thrombus formation regardless of age. Second, the mitochondria-targetable antioxidants such as SOD and MitoQ limited platelet activation and ex vivo thrombus formation in both young and middle-aged adults with prediabetes, suggesting a mito-oxidant–mediated mechanism. Third, mice mimicking early onset of glucose intolerance had increased platelet activation and greater susceptibility to in vivo thrombosis and were protected by pharmacological or genetic targeting with mitochondrial antioxidants. Finally, our mechanistic study in mice revealed that treatment with MitoQ or catalase overexpression within mitochondria lowered lipid peroxides, mitochondrial membrane hyperpolarization, and intraplatelet Ca^2+^ influx. Our data also revealed that elevation in platelet calcium levels may contribute to the regulation of mitochondrial redox status and platelet activation in the models of early onset of glucose intolerance, and increased mito-oxidants promote elevation in Ca^2+^, suggesting a complex and bidirectional interplay between calcium signaling and mito-oxidants. Together, these data provide strong evidence for the existence of a prothrombotic state in prediabetes that likely is mediated by mitochondrial oxidative stress.

This study was focused on a military veteran population. About 70% of US military veterans have either overweight or obesity (21); therefore, a high likelihood of prediabetes is expected in this cohort. Although about 25% of military veterans have established T2D (22), screening for prediabetes in active-duty military personnel is inadequate (23), and so the prevalence and consequences of prediabetes are largely unexplored in this unique population. A nationwide survey of middle-aged and older outpatients with schizophrenia seen by the Veterans Administration revealed that the incidence of prediabetes in the group was 42% (24). In a recent study (23), the percentages of patients meeting laboratory prediabetes criteria were 28.4%, 30%, and 31% in the members from the Air Force, Army, and Navy, respectively. In this context, it is important that the adverse CV events associated with military veterans’ health are reported to occur several years before diabetes is diagnosed or manifests (25), possibly during the prediabetes state. Therefore, identifying service members with prediabetes and a systematic evaluation and identification of early mechanisms are necessary to prevent future thrombotic complications in vulnerable military veterans.

Using platelets from young and middle-aged military veterans, we report that prediabetes induces platelet activation and its accumulation on collagen surfaces regardless of age and has the likelihood to mediate increased risk for CV events in military veterans with prediabetes. We further explored the mechanisms of a such prothrombotic phenotype.

Even though increased platelet activation and increased thrombotic susceptibility are well described in individuals with established T2D (26–28), the use of common antiplatelets, such as aspirin, for primary prevention is no longer routinely recommended because of the increased risk of bleeding, particularly in individuals with low to moderate CVD risk (29–32). The 2019 guidelines from the American Diabetes Association emphasized that aspirin use for primary prevention should involve individualized clinical decision-making, even among those at high risk for CVD (33). These evolving findings and recommendations underscore the need to investigate an alternative targetable pathway that potentially could be used to develop therapeutics to lower CVD risks in T2D and in prediabetes. A few studies conducted with patients with diabetes have reported increased burden of mitochondrial oxidative stress and altered mitochondrial metabolism within platelets, reflected by altered levels of antioxidants and reduced oxygen consumption (18). Yamagishi et al. (34) also showed a direct in vitro effect of hyperglycemia in causing increased platelet mitochondrial oxidative stress and platelet aggregation, with the implication that altered mitochondrial electron transport chain function contributed to the phenotype. We hypothesized that early metabolic abnormalities occur during prediabetes that contribute to premature platelet activation and a prothrombotic state. In support of this idea, we observed that platelet mitochondria in both young and middle-aged individuals with prediabetes have higher mito-oxidant levels and are hyperpolarized. This was consistent with the observed decrease in SOD2 activity in these cohorts. To test the mechanistic potential of increased mito-oxidants in causing platelet activation, we treated platelets with a SOD mimetic or MitoQ in separate experiments. Both interventions lowered mito-oxidant levels in conjunction with diminishing agonist-induced platelet activation and ex vivo thrombus formation among individuals in both age groups with prediabetes. These findings in clinical samples suggest a mito-oxidant–mediated prothrombotic state exists in individuals with prediabetes.

A relatively higher, though nonsignificant, increase in integrin activation, fibrinogen binding, and P-selectin expression observed in young individuals with prediabetes compared with older participants with prediabetes may suggest the potential for heightened mitochondrial metabolic activity at younger ages amplifying platelet reactivity. In support of this idea, we observed increased platelet mito-oxidants in young participants, but not aged participants, with prediabetes at resting state. This intriguing observation warrants validation in a larger cohort, including detailed examination of the metabolic state of platelet mitochondria. If confirmed, future studies could investigate whether mitochondrial metabolism contributes to these age-dependent differences in platelet function. Such findings may also help explain the observed clinical associations in younger individuals between prediabetes and increased risk of hospitalization for myocardial infarction (8), poorer prognosis after ischemic stroke (9), and the apparent attenuation of CV and all-cause mortality risk with advancing age in prediabetes (10). Furthermore, that prediabetes did not have an additional effect in aged individuals for several platelet activation markers, except annexin V, or for platelet thrombus formation, may also suggest that prediabetes may accelerate age-dependent mechanisms for platelet dysfunction, leading to its manifestation in younger individuals.

An intriguing observation was that although age-related increases in mito-oxidants were not evident in control participants, treatment with a SOD mimetic reduced platelet activation responses to agonists for integrin activation, fibrinogen binding, and P-selectin. This effect may reflect an intrinsic role of SOD in modulating platelet activation regardless of mitochondrial oxidative stress in aged control participants. The SOD mimetic used in our study can reduce not only mito-oxidants but cytoplasmic oxidants as well (35), which could contribute to this effect. Moreover, when platelets were treated with mitochondria-targeted MitoQ, protection from agonist-induced platelet activation was observed in only prediabetic groups but not in middle-aged normoglycemic group. This phenomenon supports a hypothesis that prediabetes-driven platelet activation may be largely modulated by mito-oxidants, whereas aging might induce platelet activation through other mechanisms.

The correlation matrix analysis allowed us to assess some meaningful clinical associations. We report the strongest associations of all platelet activation markers with HOMA-IR, insulin, FBG, and then HbA1c. Notably, minimal associations were seen between BP or lipid levels and platelet activation markers. These findings suggest the prothrombotic phenotype observed in prediabetes is likely modulated by hyperglycemia, and the association between hyperglycemia and platelet activation may exist across a larger range of FBG spanning from normoglycemia to prediabetes to diabetes. We did not include patients with diabetes in our study, because such an analysis would require a significantly larger sample size to adjust for the high prevalence of comorbid conditions commonly associated with diabetes, including established metabolic syndrome and frequent medication use in this cohort. Our study was specifically designed to examine platelet phenotype in individuals with prediabetes who have minimal or no features of metabolic syndrome or other confounding comorbid conditions that allowed us to isolate the effects of less severe hyperglycemia on platelet dysfunction. Nevertheless, findings from our study help build the rationale to direct future larger studies to comprehensively assess the relationship between FBG/hyperglycemia and platelet dysfunction.

To begin to understand the in vivo relevance of our findings in prediabetes, we used a mouse model of short-term HF feeding that did not induce obesity but caused early onset of glucose intolerance characterized by abnormal GTT results; increased random, circulating glucose concentrations; and hyperinsulinemia. This model not only phenocopied platelet hyperactivity seen in human prediabetes, but it also manifested increased susceptibility to pulmonary and carotid artery thrombosis in vivo. We also observed that treatment of mice with mitochondria-targeted antioxidants, such as a SOD mimetic, protected HF-fed mice from increased platelet mito-oxidants, platelet activation, and thrombosis of pulmonary and carotid arteries without causing bleeding or altering GTT results. We further showed that treatment with MitoQ reduced the population of total mito-oxidants, including lipid peroxides, and prevented platelet hyperactivation and in vivo thrombotic susceptibility of both pulmonary and carotid arteries in HF-fed mice, suggesting a direct role of lipid peroxides in mediating the prothrombotic phenotype. Treatment with MitoQ did not cause bleeding or alter GTT results. These findings suggest intervention with mitochondria-targetable antioxidants may protect from prothrombotic phenotype by mitigating adverse effects secondary to glucose intolerance and insulin resistance.

Furthermore, the genetic intervention to target peroxides within mitochondria by overexpression of catalase in mCAT-Tg mice also protected from increased mito-oxidant levels, including lipid peroxides, platelet activation, and susceptibility to in vivo thrombosis induced by HF diet, which is consistent with the lipid peroxide–lowering effects of MitoQ (20). These data also are consistent with protective effects of lowering platelet hydrogen peroxide on platelet activation and thrombosis that we have previously reported (11). Mechanistically, we show that increased mito-peroxide levels link Δψm, Ca^2+^ influx, platelet activation, and thrombosis. Inhibition of Ca^2+^ not only lowered platelet activation but also decreased the burden of mito-oxidants, including lipid peroxide. Notably, calcium and oxidative stress appear to influence each other in a bidirectional manner: when oxidative stress promotes calcium influx, the elevated calcium levels appeared to exacerbate oxidative stress (36, 37). Consistent with these ideas, our data suggest enhanced mitochondrial oxidative stress might be partly mediating platelet activation via calcium dysregulation where increased intracellular Ca^2+^ further exacerbates the mito-oxidant burden. This idea can be expanded in future studies using platelet-specific expression models. Our data also suggest an important role of mitochondrial Ca^2+^ in sustaining the increase in Δψm, by perhaps driving Ca^2+^-sensitive mitochondrial metabolism, explaining why blocking mitochondrial Ca^2+^ uptake diminishes Δψm to a greater extent than extracellular Ca^2+^ chelation. Using conditional platelet SOD2-deficient mice, we previously reported dependence of platelet calcium levels, mitochondrial hyperpolarization, and PS exposure on mito-oxidants in a model of aging (13). Thus, our mechanistic findings are consistent with prior work demonstrating that a sustained increase in intraplatelet calcium has the potential to alter Δψm and mediate PS exposure (38–40).

We also considered that the relationship between Δψm and mito-oxidant generation is dynamic and not necessarily unidirectional. Under normal conditions, optimal electron leak from the electron transfer chain maintains a moderate Δψm that supports efficient ATP synthesis. However, mitochondrial hyperpolarization can slow electron transport, increasing electron leakage from resident complexes I and III and thereby elevating superoxide, hydrogen peroxide, and lipid peroxidation (41–43). Partial inhibition of ATP synthase can also reduce proton flux through complex V and maintain elevated Δψm while increasing mito-oxidant generation, as reported to occur in platelets from patients with sickle cell disease (44). Our group and others have also reported coexistence of increased mito-oxidants and lipid peroxidation in the presence of hyperpolarized mitochondria in platelets from aged mice and humans or due to hyperglycemia (12, 13, 34). Additionally, hyperpolarization of mitochondria could be related to a regulatory effect of oxidants on Ca^2+^ levels. This idea is consistent with our observation of reduced platelet Ca^2+^ in association with lowered Δψm upon MitoQ treatment of mice fed an HF diet or in mCAT-Tg mice fed an HF diet. It is also important to note that mito-oxidants’ generation is not solely dependent on Δψm (45). Localized lipid peroxidation (46) and limited SOD2 activity may also amplify these effects. Thus, the mitochondrial phenotype may depend on several factors such as the degree of hyperpolarization, Ca^2+^ dysregulation, lipid peroxidation, presence of antioxidant enzymes, and it may vary in different disease states. Fully understanding the specific mechanisms that drive increased mito-oxidant levels in the presence of hyperpolarized mitochondria in prediabetes requires extensive additional work that will be the focus of future studies.

Taken together, using 2 pharmacological approaches and a genetic approach, we demonstrate that targeting mito-oxidants — specifically peroxides — is protective and represents a key mechanism that contributes to the prothrombotic effects of an HF diet. Our findings in the preclinical model of early onset of glucose intolerance provides a rationale for considering GC4419 or MitoQ as a potential treatment. GC4419 can be administered as a drug and has been used in cancer models to target oxidative stress (19, 47). MitoQ has also been tested and shown to protect from endothelial dysfunction in older people (48, 49).

Our study has some limitations. First, considering the small sample size and the cross-sectional nature of the human study, we are unable to evaluate associations with long-term clinical outcomes. Future studies should consider longitudinal follow-up to better define the trajectory of the prothrombotic phenotype in this population and the time course of vascular events. In addition, considering the small study size, we cannot exclude the potential for residual confounding factors. For example, BP was significantly higher among the middle-aged participants with prediabetes, and HDL-cholesterol was lower among the young participants with prediabetes compared with their age-matched controls. Last, due to predominance of male participants in the Veterans Administration system, the effect of sex on human prediabetes could not be evaluated. Nevertheless, our study has several strengths, including the well-defined inclusion and exclusion criteria, the inclusion of young and middle-aged control cohorts, a comprehensive approach of using human and animal models for examining platelet activation and susceptibility to ex vivo and in vivo thrombosis, and the use of pharmacological and genetic approaches to address underlying mechanisms.

In conclusion, we report the existence of a prothrombotic phenotype among young and middle-aged adults with prediabetes. Through ex vivo and in vivo studies, we have identified mito-oxidants or peroxides as a key mechanism by which the prothrombotic state develops in prediabetes. The clinical impact of our findings is driven by the clearly documented associations between prediabetes and CVD events in the prospective studies and the recent use of mitochondria-targeted antioxidants in patients with cancer and in patients who are elderly (19, 47–49). Additional research is needed to evaluate whether targeting platelet hyperactivity will reduce the risk of CV events in those with prediabetes.

## Methods

### Sex as a biological variable.

Our study in humans included men and women. The animal study examined male and female mice, and similar findings are reported for both sexes.

### Flow cytometry.

Washed platelets were used for flow cytometry. Activation of integrin α_IIb_β3 was evaluated by quantifying PAC1 and JON/A binding in human and mouse platelets, respectively, and fibrinogen binding was measured in human platelets. The α-granule secretion was measured by surface expression of P-selectin (CD62P), PS exposure by annexin V binding, and mito-oxidants and lipid peroxidation by MitoSOX and BODIPY 581/591 C11 fluorescent dyes, respectively (13, 50, 51). Washed platelets (1 × 10^8^/mL) were incubated either with 5 μL of PE-conjugated PAC1 (1:3 vol/vol; BioLegend), PE-conjugated JON/A (1:3 vol/vol; Emfret Analytics), FITC-conjugated chicken anti-human fibrinogen (1:5, vol/vol; Diapensia), FITC-conjugated rat anti-human or anti-mouse CD62P (1:3 vol/vol; BioLegend) antibody, Annexin V-Alexa Fluor 647 (1:1; BioLegend), 2.5 μM MitoSOX (Thermo Fisher Scientific), or 10 μM BODIPY 581/591 C11 (Thermo Fisher Scientific) activated with human thrombin alone or in combination with convulxin, for 10 minutes at 37°C, and all the tubes containing antibodies were fixed in 1% paraformaldehyde. For PS exposure, a binding buffer (100 μL) was added. All the samples were diluted 10-fold in 1× PBS and analyzed using an LSR Violet flow cytometer (Becton Dickinson).

To evaluate Δψm, washed platelets were stained with 500 nM TMRM (Invitrogen) for 15 minutes in the presence or absence of 5 or 10 μM FCCP (52), an uncoupler that activates proton conductance. In some experiments, platelets were treated with GC4419 (25 or 50 μM), MitoQ (5 or 10 μM), BAPTA-AM (150 μM), Ru360 (150 μM), or respective vehicle control or buffer (bicarbonate buffer saline for GC4419 and Tyrode for others) for 30 minutes prior to assay. These data are presented as geometric MFI.

### Measurement of intraplatelet calcium.

Intraplatelet calcium levels or flux (mobilization and uptake) were measured in washed platelets using a previously described method with minor modifications (13). Briefly, washed platelets (1 × 10^8^/mL) were incubated with 4.5 μM Fluo-4 for 30 minutes in the dark at 37°C, then washed and resuspended in Tyrode buffer containing 2 mM CaCl_2_, and a baseline signal was acquired using a BD Accuri C6 flow cytometer for 60 seconds, followed by addition of thrombin to activate platelets. The fluorescent signals were acquired over time. Baseline signal and cumulative signals up to 2 minutes after adding thrombin were compared between the groups. In some experiments, platelets were treated with 150 μM of either BAPTA-AM or Ru360 for 30 minutes at 37***°***C prior to the assay (40, 53).

### Experimental thrombosis.

We used 2 experimental models to assess susceptibility to thrombosis in vivo. To assess the susceptibility to pulmonary thrombosis, anesthetized mice were infused with 0.5 μg/g collagen (Chronolog) retro-orbitally, which results in platelet activation–dependent pulmonary thrombosis leading to death (13, 17). After infusion of collagen, mice were monitored for up to 10 minutes for symptoms of thrombotic shock, including bradycardia, irregular breathing, and death (defined as the absence of a heartbeat for longer than 60 seconds). If death did not occur within 10 minutes, the experiment was terminated at that time and mice were sacrificed. Carotid artery thrombosis was induced by photochemical injury as described previously (11, 50). Briefly, the right common carotid artery was transilluminated with a 1.5 mV, 540 nm green laser (Melles Griot). Rose bengal (25 mg/kg) was injected via a femoral vein catheter. Carotid artery blood flow was monitored with a Doppler flow probe for up to 90 minutes after injury or until a stable occlusion developed. Stable occlusion was defined as the time at which blood flow remained absent for at least 10 minutes.

Details of methods such as measurements for insulin and blood glucose, GTT, platelet isolation, platelet adhesion assay, expression and activity of SOD2, and tail bleeding assays are provided in Supplemental Methods.

### Statistics.

All data were analyzed using GraphPad Prism, version 10.4.1. Normality and log-normality were assessed using the D’Agostino and Pearson test if sample size was greater than 6, and the Shapiro-Wilk test was used if sample size was 6 or fewer. For datasets showing normal or log-normal distributions, the 2-tailed unpaired *t* test was used for 2-group comparisons, and the 2-way ANOVA was used for multiple groups with Tukey’s multiple-comparison test. Some data were also analyzed using 1-way ANOVA with Dunn’s analysis, whereby, within each cohort, treatment with multiple doses of drugs were compared with the control treatment. For data not in a normal distribution, the Kruskal-Wallis test was used for multiple group comparisons. Results of time-dependent tests such as the GTT were analyzed with mixed effect analysis with Šídák’s multiple comparisons. A correlation matrix analysis with the Spearman correlation was performed to analyze associations among age, BMI, FBG, HbA1c, insulin, HOMA-IR, lipids, BP, and platelet activation parameters. A *P* value less than 0.05 was considered significant.

### Study approval.

Our human cohort study complied with all relevant ethical regulations and was approved by the University of Iowa Institutional Review Board (approval 202002028). Written informed consent was received prior to participation. Mice were housed in the animal facility of the University of Iowa, experimental protocols were approved by the University of Iowa Institutional Animal Care and Use Committee (approval 2390203), and all relevant ethical regulations were followed, including for experimental thrombosis protocols. Further details about human participants and mice are provided in Supplemental Methods.

### Data availability.

Values for all data points in the graphs are provided in the Supporting Data Values file. All remaining materials and methods are explained in Supplemental Methods. Any data and materials that can be shared will be released via a material transfer agreement.

## Author contributions

AA and PY designed and conducted the experiments, interpreted the results, and helped write the manuscript. MJ and JS conducted some of the experiments. KG assisted with participant screening, recruitment, and maintaining data in RedCap. DRS provided critical reagents and assisted with experimental design and interpreting results. EDA provided conceptual guidance that informed study design. DJ helped with patient recruitment and participated in experimental design and data interpretation. SD conceived the idea, directed the project, designed the experiments, interpreted the results, and helped write the manuscript. All authors assisted with the preparation and editing of the manuscript.

## Funding support

This work is also the result of NIH funding, in part, and is subject to the NIH Public Access Policy. Through acceptance of this federal funding, the NIH has been given a right to make the work publicly available in PubMed Central.

- Office of Research and Development and Department of Veterans Affairs (grants I01CX001932 and 2I01BX007087 to SD).
- NIH National Institute of Allergy and Infectious Diseases (grant AI162778 to SD).
- NIH U01AI184289 to DRS.
- NIH National Heart, Lung, and Blood Institute (grants HL168630 to SD, HL134738 to DJ, and HL141783 to EDA).
- NIH National Institute of Diabetes and Digestive and Kidney Diseases (grant DK133240 to DJ).
- NIH National Cancer Institute (grants CA217797, CA302572, CA244091, and P30 CA086862 to DRS).
- Institute for Clinical and Translational Science at the University of Iowa, CTSA program grant UM1TR004403.
- American Heart Association Postdoctoral Fellowship to AA (award 24POST1195019; (https://doi.org/10.58275/AHA.24POST1195019.pc.gr.190825).

## Supplementary Material

Supplemental data

Unedited blot and gel images

Supporting data values

## Figures and Tables

**Table 1:**
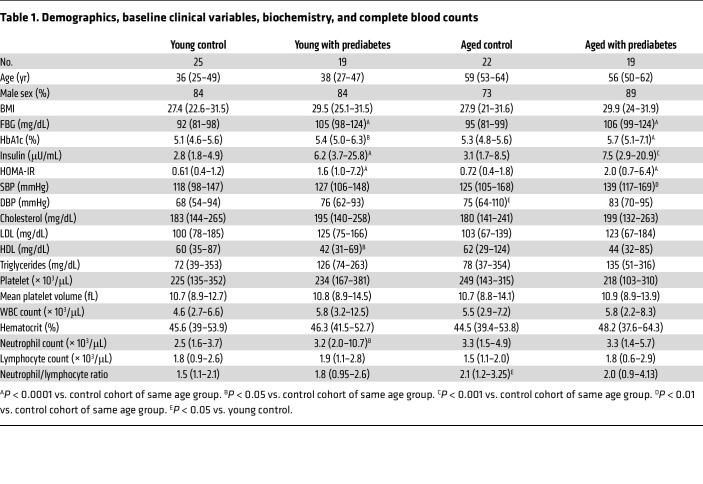
Demographics, baseline clinical variables, biochemistry, and complete blood counts

## Version 1 12/23/2025

In-Press Preview

## Version 2 02/16/2026

Electronic publication
