# Supplementary material for: Mitochondrial oxidants promote platelet activation and thrombotic susceptibility in prediabetes
Source: J Clin Invest. 2025 Dec 23;136(4):e195662. doi: 10.1172/JCI195662 (PMC12904718; doi:10.1172/JCI195662)
Supplement: Supplemental data [file jci-136-195662-s246.pdf]

SUPPLEMENTAL MATERIAL

Title: Mitochondrial Oxidants Promote Platelet Activation and Thrombotic Susceptibility in Prediabetes

SUPPLEMENTARY METHODS

Human subjects

Veterans were recruited from the Iowa City VA Healthcare System (HCS). Two age-groups with prediabetes were recruited: 18-50 years for the young prediabetes group and 51-64 years for the middle-aged prediabetes group. Prediabetes was defined as FBG of 100-125 mg/dL and/or HbA1c 5.7-6.4 %, as per American Diabetes Association guidelines (1). We also recruited healthy young (18-50 years) and middle-aged (51-64 years) Veterans concurrently as controls. The following exclusion criteria were applied to all groups: a) use of antiplatelet drugs, anticoagulants or non-steroidal anti-inflammatory drugs in the previous 12 days, b) currently taking medications for diabetes, c) pre-existing conditions of thromboembolic disease or a bleeding disorder, d) active cancer or past history of cancer, e) surgery in the past 2 months, f) currently taking oral contraceptives or hormone replacement therapy, g) pregnant or lactating women, h) overseas travel in the past 10 days that could increase DVT risk, and i) smokers. Additional exclusion criteria used for healthy subjects, was history of cardiovascular or metabolic disease. Written informed consent was obtained from all eligible participants. All protocols were approved by the institutional review board of the University of Iowa and the Iowa City VA HCS. Blood samples were collected from subjects after an overnight fast. The body mass index (BMI) and blood pressure was recorded on the day of recruitment and routine blood tests for complete blood cell count (CBC), FBG, HbA1c, and lipid panel were obtained.

Mice

All animal studies were approved by the institutional animal care and use committee of the University of Iowa. C57BL6/J mice were obtained from the Jackson Laboratory (Bar Harbor, Maine, Strain #:000664) and were bred and maintained in the animal facility at the University of Iowa. Transgenic male mice overexpressing mitochondria-targeted catalase (mCAT-Tg) were purchased from Jackson Laboratory (Strain #:016197) and bred in our facility with female C57BL6/J mice to generate mCAT-Tg and wild-type (WT) control littermates. Mice were fed either control chow or high fat (HF) diet containing 60% Kcal from fat, for 2 weeks starting at 6 weeks of age. After one week on diets, the mice either received GC4419, at a dose of 10 mg/Kg daily via intraperitoneal injection for 1 week (2), or vehicle control (Bicarbonate Buffer Saline). In a second set of experiments, mice received either the antioxidant, MitoQ at a dose of 1 mg/Kg, or vehicle buffer. Male and female mice were included in the study design.

Insulin, blood glucose and glucose tolerance test

We measured plasma insulin levels in humans and mice using commercially available ELISA kits (Alpco, Cat# 80-INSHU-CH01 and Crystal Chem, Cat# 90080 respectively), following the manufacturer's instructions. A glucose tolerance test (GTT) was performed to evaluate glucose homeostasis in mice. After overnight fasting, a baseline glucose level was measured using a glucometer (OneTouch® UltraMini® meter) in blood collected via tail vein. Mice were then injected intraperitoneally (IP) with Dextrose (1 g/kg) and blood glucose levels were monitored at 5, 15, 30, 60, and 120-minutes post injection (3). Non-fasting blood glucose was also measured in randomly fed mice.

Platelet isolation

Human and mouse platelets were prepared from blood collected in tubes containing 3.2% sodium citrate (4). Blood was initially centrifuged at 100 x *g* for 10 minutes at room temperature to collect platelet rich plasma (PRP). After addition of 1 μM prostaglandin E_1 (PGE_1, Sigma Corporation), human PRP was centrifuged at 1000 x *g* and mouse samples were centrifuged at 800 x *g* for 10 minutes at room temperature. The platelet pellet was collected and washed in Tyrode's buffer (134 mM NaCl, 2.9 mM KCl, 0.34 mM Na_2HPO_4, 12 mM $NaHCO_3$, 20 mM HEPES buffer, 1 mM $MgCl_2$, 5.5 mM glucose) containing 1 μM PGE_1 at pH 6.5 for human and pH 7.35 for murine platelets. Finally, washed platelets from both humans and mice were centrifuged at 1000 x *g* and 800 x *g* respectively for 10 minutes at room temperature and the pellet was suspended in Tyrode's buffer at pH 7.35 containing 0.35% bovine serum albumin.

Microfluidics-based platelet adhesion on collagen matrix under arterial shear

Platelet thrombus growth on collagen matrix was measured in a microfluidic BioFlux™ flow chamber (Fluxion Biosciences) (4). High-shear plates were coated with 50 μg collagen (Chrono-log) and blocked with 1% BSA. Platelets (1 x 10^8/mL) labeled with calcein-green (2.5 μg/mL) were perfused over collagen at a physiological arterial shear rate (2000 s^{-1}) for 5 min. Total thrombi area at 5 min was calculated by average accumulation of platelets in 5 representative fields and analyzed using ImageJ (NIH).

Expression and activity of SOD2

Platelets were first lysed in RIPA buffer for western blot and in sodium phosphate buffer with mild detergents for SOD activity assay in the presence of protease and phosphatase inhibitors, and protein was quantified by bicinchoninic acid (BCA) analysis. Anti-SOD2 antibody [developed at the University of Iowa] (5), and Anti-β actin (sc-477788H10D10: Cell Santa Cruz) were used as primary antibody and blots were incubated with HRP-linked secondary antibody for 1 hour at room temperature. Densitometry of images was analyzed using ImageJ and the ratio of SOD2/β-actin of each sample is presented. The activity of SOD2 was determined using an assay as described (2), with an assay kit purchased from Cayman Chemical (Cat# 706002, Ann Arbor, MI) and normalized to protein content, following the manufacturer's instructions. For the assay, 50 µg of platelet protein lysates were incubated with xanthine oxidase and tetrazolium salt. Total SOD activity was determined as inhibition of superoxide anion production over 30 minutes. To specifically determine SOD2 activity, SOD1 activity was inhibited by addition of 3 mM potassium cyanide.

Tail bleeding assay

Mouse tail clip bleeding assay was performed as previously described (6). Briefly, mice were anesthetized with isoflurane and placed on a heating pad at 37 °C. The tip of the tail was transected 3 mm with a sharp razor blade and immersed in a 50 ml tube containing pre-warmed saline at 37 °C. Bleeding time was measured as the time taken for the blood flow to stop for at least 30 seconds.

References

1. American Diabetes A. 2. Classification and Diagnosis of Diabetes: Standards of Medical Care in Diabetes-2020. *Diabetes Care.* 2020;43(Suppl 1):S14-S31.
2. Sonkar VK, Eustes AS, Ahmed A, Jensen M, Solanki MV, Swamy J, et al. Endogenous SOD2 (Superoxide Dismutase) Regulates Platelet-Dependent Thrombin Generation and Thrombosis During Aging. *Arterioscler Thromb Vasc Biol.* 2023;43(1):79-91.
3. Pedro PF, Tsakmaki A, and Bewick GA. The Glucose Tolerance Test in Mice. *Methods Mol Biol.* 2020;2128:207-16.
4. Sonkar VK, Kumar R, Jensen M, Wagner BA, Sharathkumar AA, Miller FJ, Jr., et al. Nox2 NADPH oxidase is dispensable for platelet activation or arterial thrombosis in mice. *Blood Adv.* 2019;3(8):1272-84.
5. Mapuskar KA, Flippo KH, Schoenfeld JD, Riley DP, Strack S, Hejleh TA, et al. Mitochondrial Superoxide Increases Age-Associated Susceptibility of Human Dermal Fibroblasts to Radiation and Chemotherapy. *Cancer Res.* 2017;77(18):5054-67.
6. Patel RB, Dhanesha N, Sutariya B, Ghatge M, Doddapattar P, Barbhuyan T, et al. Targeting Neutrophil alpha9 Improves Functional Outcomes After Stroke in Mice With Obesity-Induced Hyperglycemia. *Stroke.* 2023;54(9):2409-19.

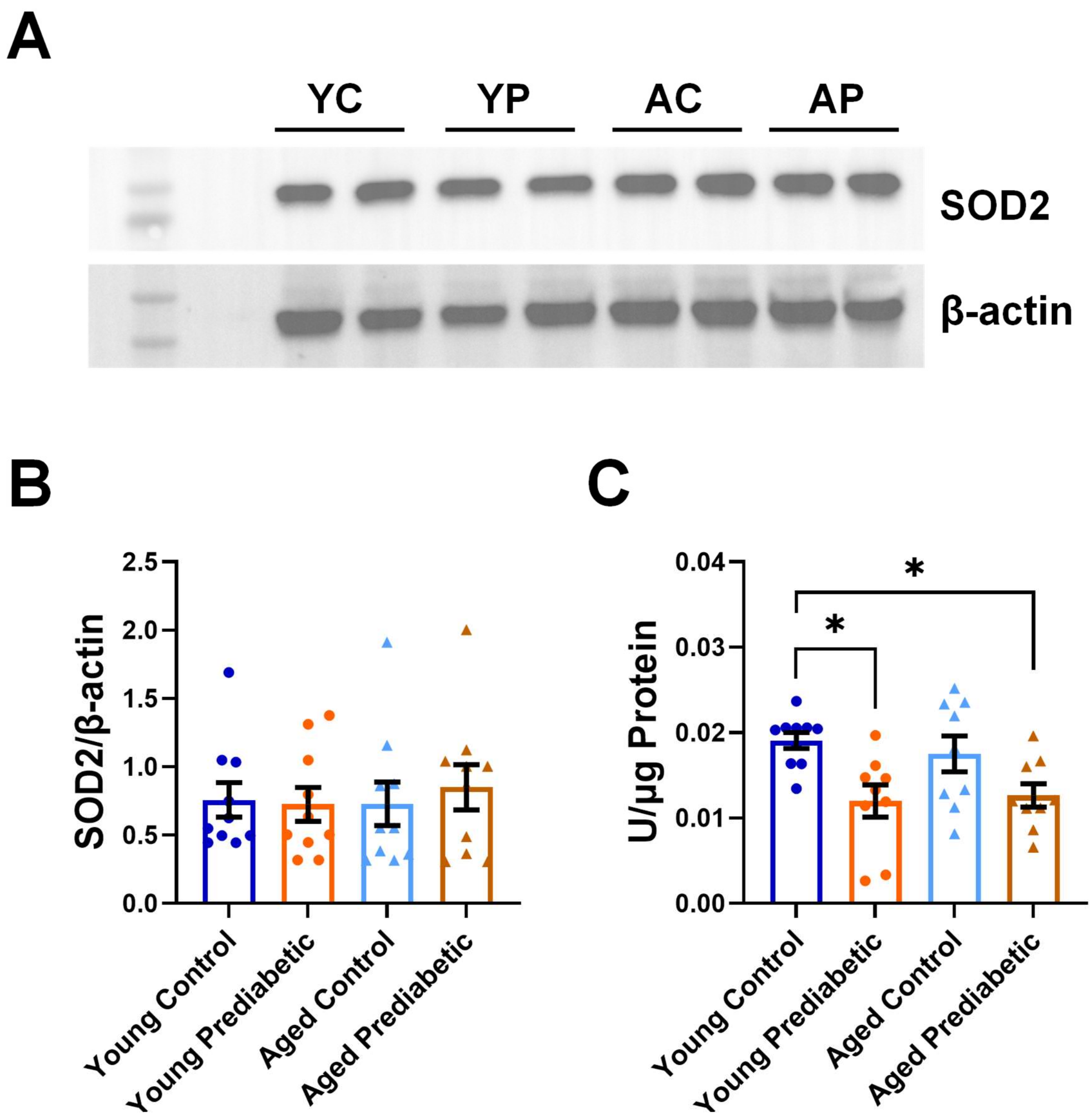

Supplemental Figure 1. SOD2 expression and activity in human platelets. Bead purified platelets were prepared from young and middle-aged control or prediabetic Veterans. (**A**) Representative image of immunoblot of SOD2 protein expression in platelets from young control and young prediabetic (YC and YP respectively) or aged control and aged prediabetic Veterans (AC and AP respectively). (**B**) Quantification of SOD2 protein expression using Image J. (**C**) SOD2 activity. Data are presented as mean ± SE and analyzed using two-way ANOVA with Tukey's analysis for multiple comparisons. N = 9 -10 per group. *P < 0.05.

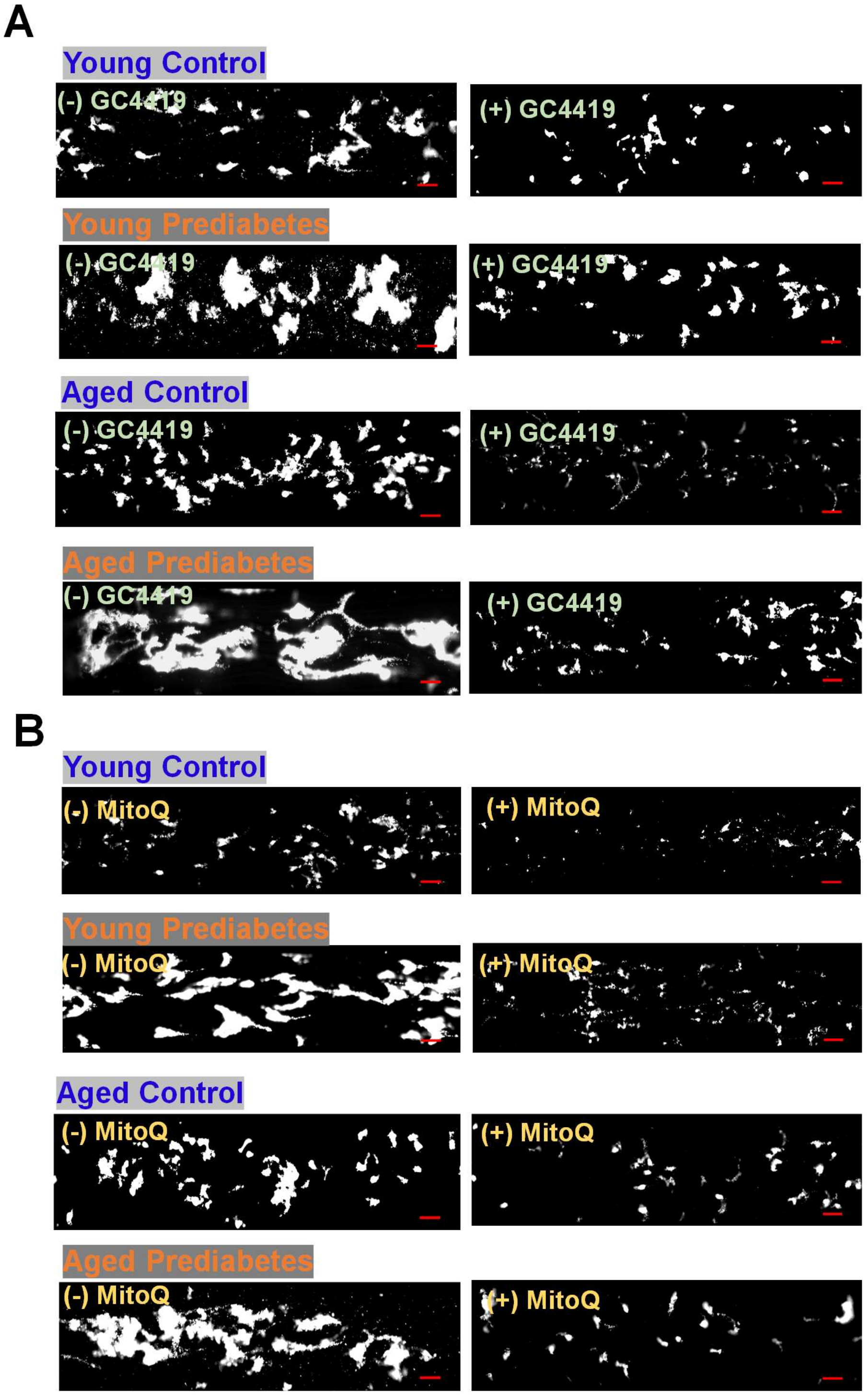

Supplemental Figure 2. Representative images of platelet accumulation under arterial shear stress. Washed platelets were superfused over collagen coated microfluidic wells under arterial shear. Accumulation of platelets are shown after 5 min. in the respective groups with prior incubation of platelets (**A**) with vehicle buffer (-) GC4419 or with (+) GC4419, and (**B**) with vehicle buffer (-) MitoQ or with (+) MitoQ. Scale bar = 100 µM.

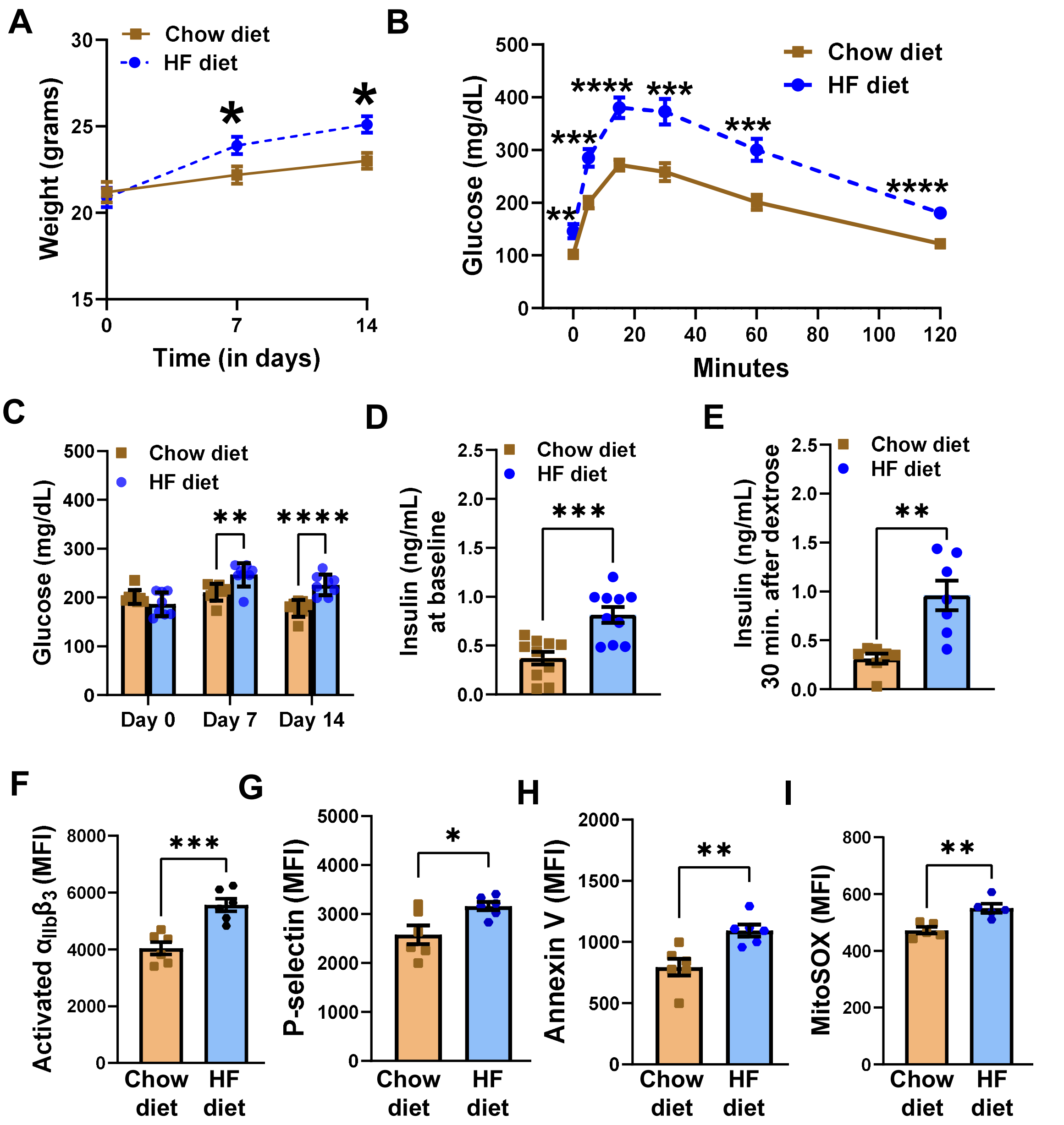

Supplemental Figure 3. Mice fed short-term high fat diet exhibit glucose intolerance, and exhibit platelet activation and enhanced mito-oxidants within platelets. C57BL6J mice were fed a chow or high fat (HF) diet for two weeks. (**A**) weight gain over 14 days, (**B**) glucose tolerance test after dextrose injection (1 g/kg IP), (**C**) Random blood glucose, (**D**) plasma insulin after 2 weeks of dietary treatment, (**E**) plasma insulin 30 min after dextrose injection (IP). Washed platelets were prepared for quantifying (**F**) $\alpha_{IIb}\beta3$ activation, (**G**) P selectin expression, (**H**) annexin V binding, and (**I**) mitochondrial oxidants, activated with 0.05 U/mL thrombin for (F and G) and with 0.05 U/mL thrombin and 50 ng/mL convulxin for (H and I), and analyzed via flow cytometry. Data are presented as mean ± SE and analyzed using unpaired t-test. N = 10 per group for (A), 21 per group for (B), 8 per group for (C), 10 per group for (D), 7 per group for (E), 6 per group for (F-H) and 5 per group for (I). *P < 0.05, **P < 0.01, ***P < 0.001, ****P < 0.0001.

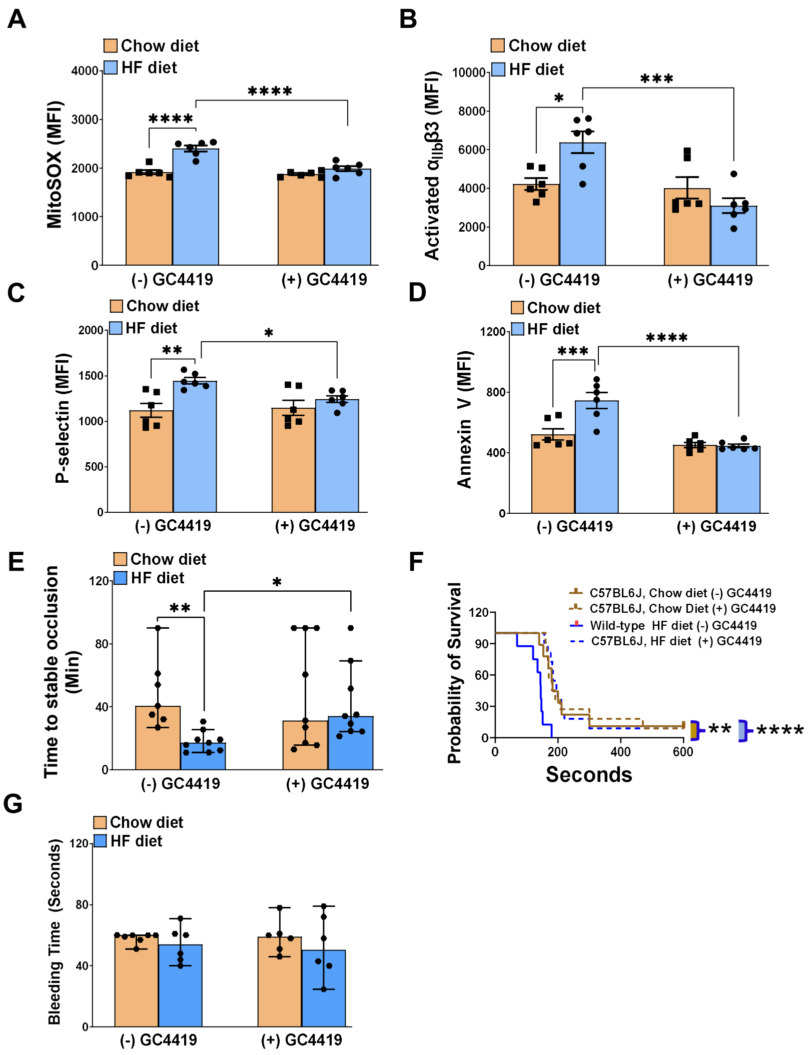

Supplemental Figure 4. In vivo treatment with GC4419 protected mice fed high fat diet from enhanced generation of platelet mito-oxidants, platelet activation and increased susceptibility to carotid artery and pulmonary thrombosis. C57BL6/J mice fed chow or high fat (HF) diet for two weeks were treated daily with (+) GC4419 (10 mg/Kg daily, IP) or vehicle buffer (-) GC4419 after one week on the diet. Washed platelets were prepared for quantifying (**A**) mitochondrial oxidants, (**B**) $\alpha_{IIb}\beta3$ activation, (**C**) P selectin expression, and **D.** Annexin V binding, and activated with 0.05 U/mL thrombin and 50 ng/mL convulxin for A & D, and with 0.05 U/mL thrombin for (B and C) and analyzed via flow cytometry. (**E**) Time to stable occlusion of the carotid artery following photochemical injury. (**F**) Time to death after infusion with 0.5 µg/g collagen, shown as survival curve. (**G**) Tail bleeding time. Data for (A to D) and (G) are presented as mean ± SE and analyzed using two-way ANOVA with Tukey's test for multiple group comparisons (N = 6-7 per group). Data for (E) are presented as median with 95% CI and analyzed by Kruskal-Wallis test with Dunn's post hoc test for multiple group comparisons (N = 7-9 per group). Data for (F) is analyzed using Log-rank (Mantel-Cox) test: Comparison between HF groups (-) or (+) GC4419 is shown with blue bracket with blue fill and between chow and HF fed (-) GC4419 groups is shown as blue bracket with brown fill. N = 8-11 per group. *P < 0.05, **P < 0.01, ***P < 0.001, ****P < 0.0001.

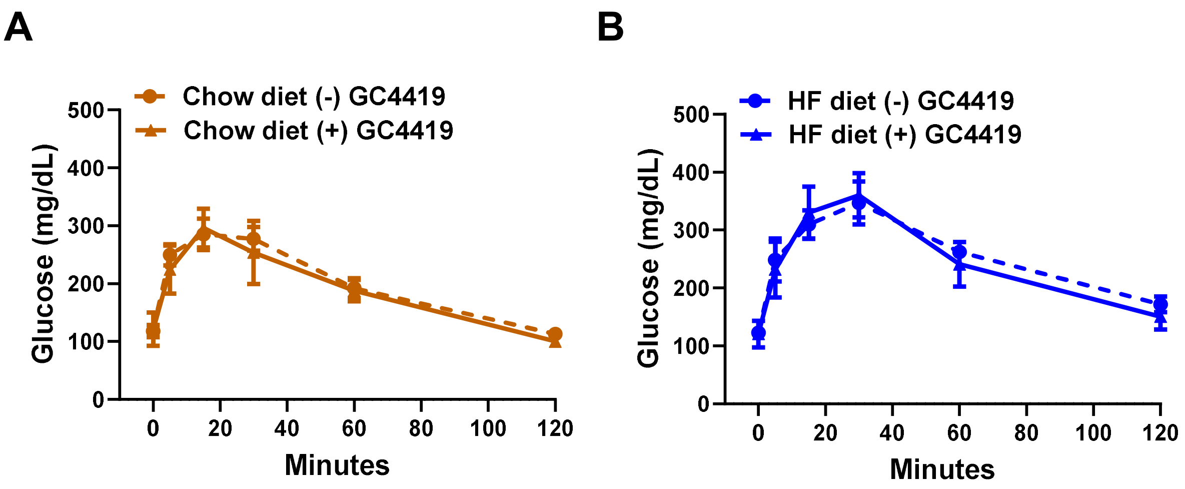

Supplemental Figure 5. In vivo treatment with GC4419 did not alter glucose tolerance in mice with short-term glucose intolerance. C57BL6/J mice fed chow or high fat (HF) diet for two weeks were treated daily with (+) GC4419 (10 mg/Kg daily, IP) or vehicle buffer (-) GC4419 after one week on the diet. Glucose tolerance test after dextrose injection (1 g/kg IP) in mice fed either (**A**) chow diet or (**B**) HF diet. Data are presented as mean±SE and analyzed with mixed effect analysis with Sidak's multiple comparisons. N = 5-6 in each group.

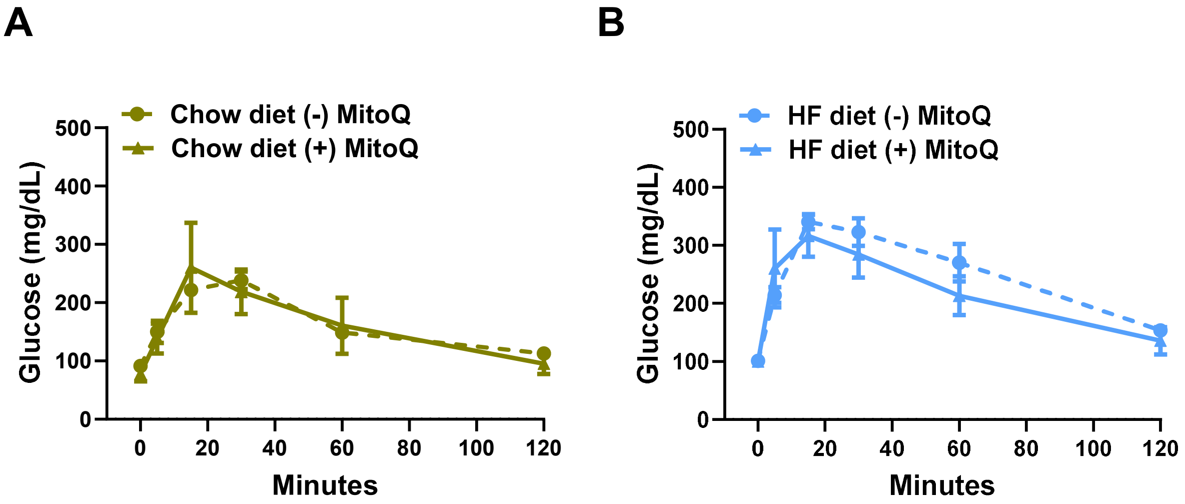

Supplemental Figure 6. In vivo treatment with MitoQ did not alter glucose tolerance in mice short-term glucose intolerance. C57BL6/J mice fed chow or high fat (HF) diet for two weeks were treated daily with (+) MitoQ (10 mg/Kg daily, IP) or vehicle buffer (-) MitoQ after one week on the diet. Glucose tolerance test after dextrose injection (1 g/kg IP) in mice fed either (**A**) chow diet or (**B**) HF diet. Data are presented as mean±SE and analyzed with mixed effect analysis with Sidak's multiple comparisons. N = 5-7 in each group.

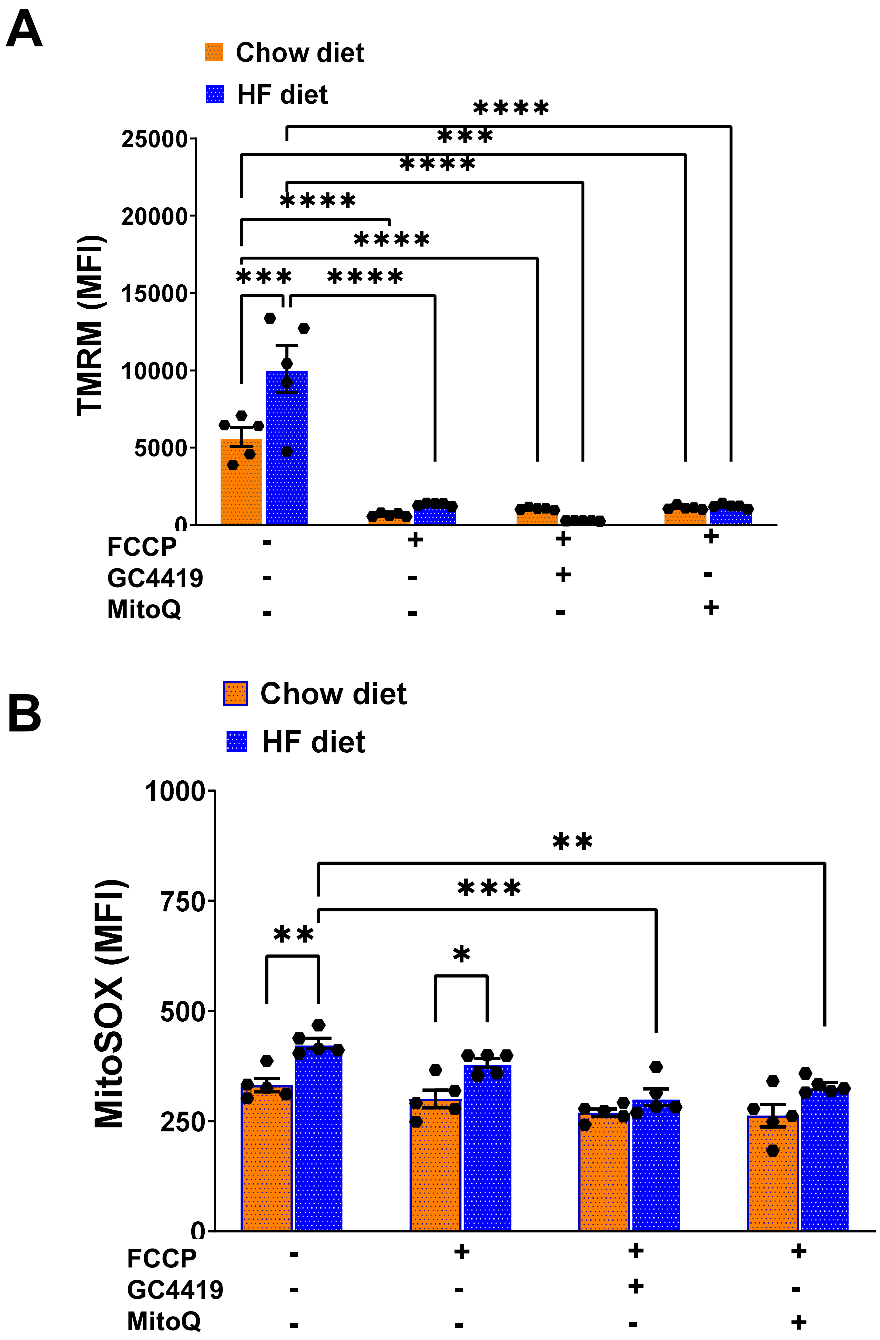

Supplemental Figure 7. Accumulation of MitoSOX in mitochondria is independent of mitochondrial membrana potential. Washed platelets were prepared from C57BL6/J mice fed chow or high fat (HF) diet for two weeks, preincubated with either vehicle buffer or 10 µM FCCP, 50 µM GC4419 or 10 µM MitoQ before performing flowcytometry for **(A)** TMRM or **(B)** MitoSOX fluorescence. Data are presented as mean ± SE and analyzed using two-way ANOVA with Tukey's test for multiple group comparisons (N = 5 per group). $^{*}P < 0.05$, $^{**}P < 0.01$, $^{***}P < 0.001$, $^{****}P < 0.0001$.

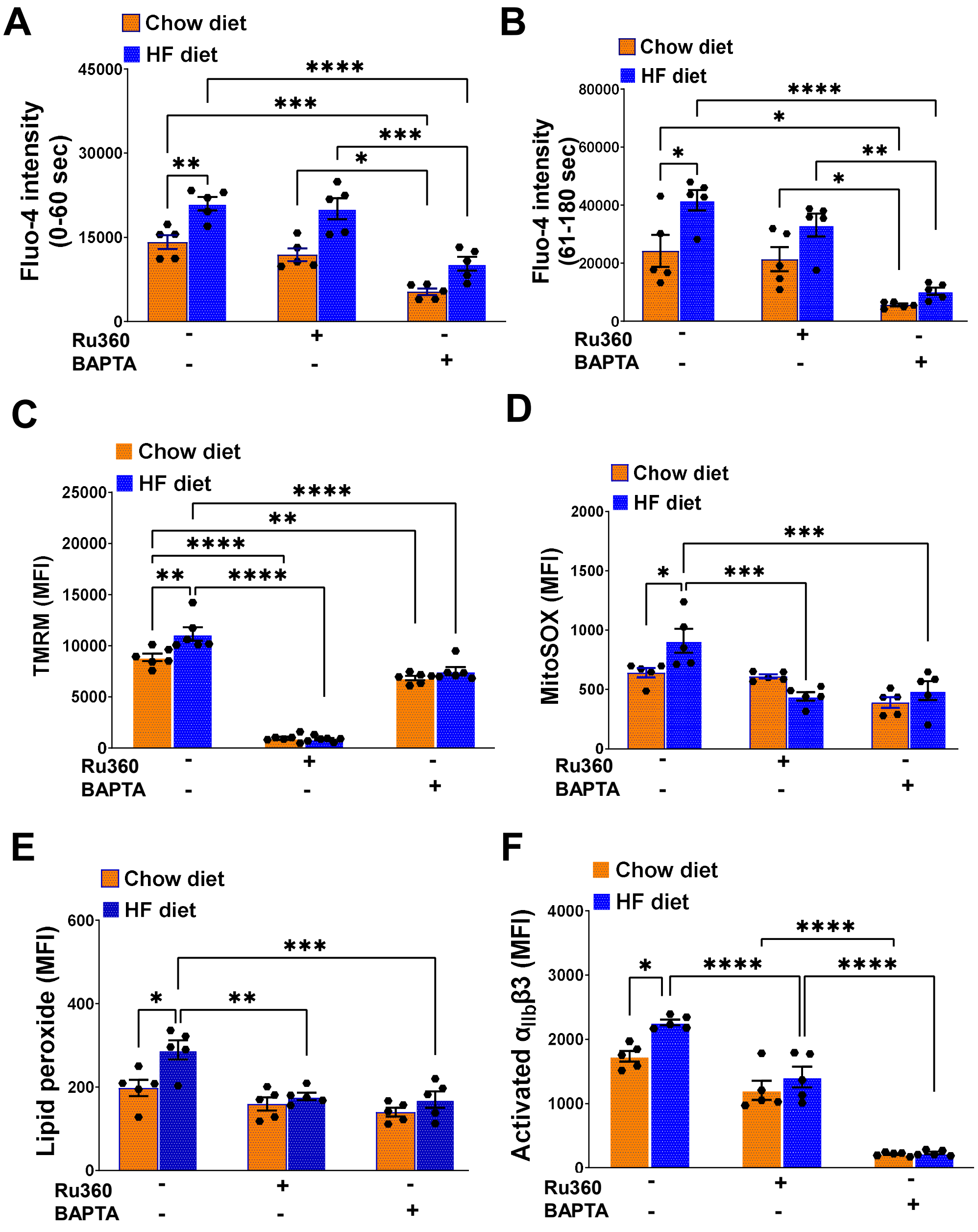

Supplemental Figure 8. Impact of altering extracellular Ca^{2+} or mitochondrial Ca^{2+} uptake on mitochondrial hyperpolarization, accumulation of mito-oxidants, lipid peroxides, and integrin activation. Washed platelets were prepared from C57BL6/J mice fed chow or high fat (HF) diet for two weeks, treated with 150 µM of Calcium chelator BAPTA-AM or mitochondrial calcium uniporter (MCU) inhibitor Ru360 for 30 min at 37 °C. Flow cytometric analysis was then performed to measure Ca^{2+} flux with Fluo-4 intensity at (**A**) baseline or (**B**) with thrombin activation (0.05 U/mL), (**C**) TMRM fluorescence, (**D**) MitoSOX fluorescence and (**E**) lipid peroxides after activation with 0.05 U/mL thrombin and 50 ng/mL convulxin, and **(F)** integrin activation with 0.05 U/mL thrombin. Data are presented as mean ± SE and analyzed using two-way ANOVA with Tukey's test for multiple group comparisons (N = 5-6 per group). *P < 0.05, **P < 0.01, ***P < 0.001, ****P < 0.0001.

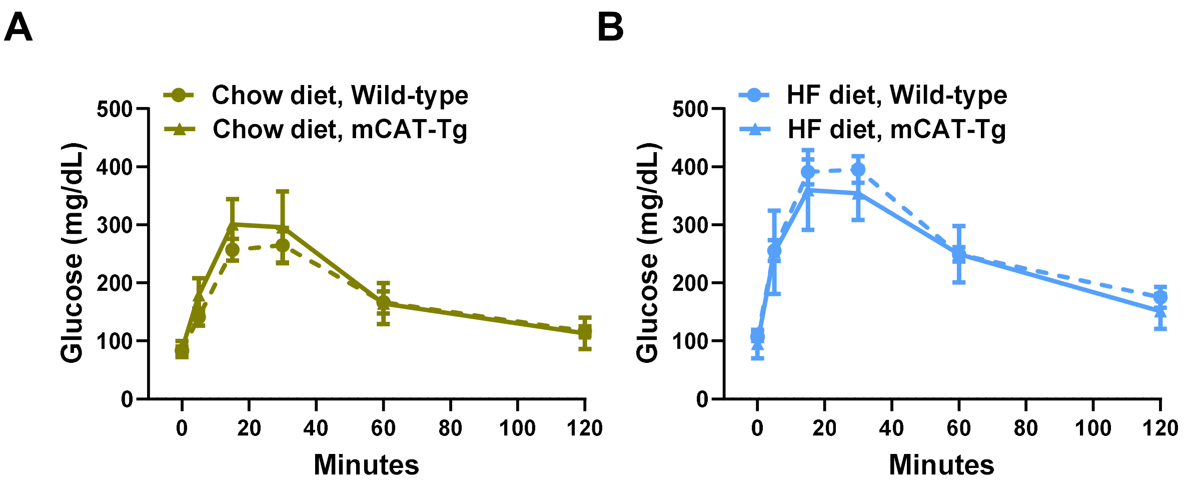

Supplemental Figure 9. Mice overexpressing mitochondria catalase develop glucose intolerance when fed HF diet. Mice overexpressing catalase in mitochondria (mCAT-Tg) or wild-type littermates were fed chow or high fat (HF) diet for two weeks. Glucose tolerance test after dextrose injection (1 g/kg IP) in mice fed either (**A**) chow diet or (**B**) HF diet. Data are presented as mean±SE and analyzed with mixed effect analysis with Sidak's multiple comparisons. N = 6-8 in each group.
