# Supplementary material for: Mitochondrial oxidants promote platelet activation and thrombotic susceptibility in prediabetes
Source: J Clin Invest. 2025 Dec 23;136(4):e195662. doi: 10.1172/JCI195662 (PMC12904718; doi:10.1172/JCI195662)
Supplement: Unedited blot and gel images [file jci-136-195662-s247.pdf]

Full unedited blot /gel for Figure 2B

Blot-5 (12-21-2024) Used as representative image in the manuscript for Figure 2B

Bio-rad ChemiDoc™ MP imaging system

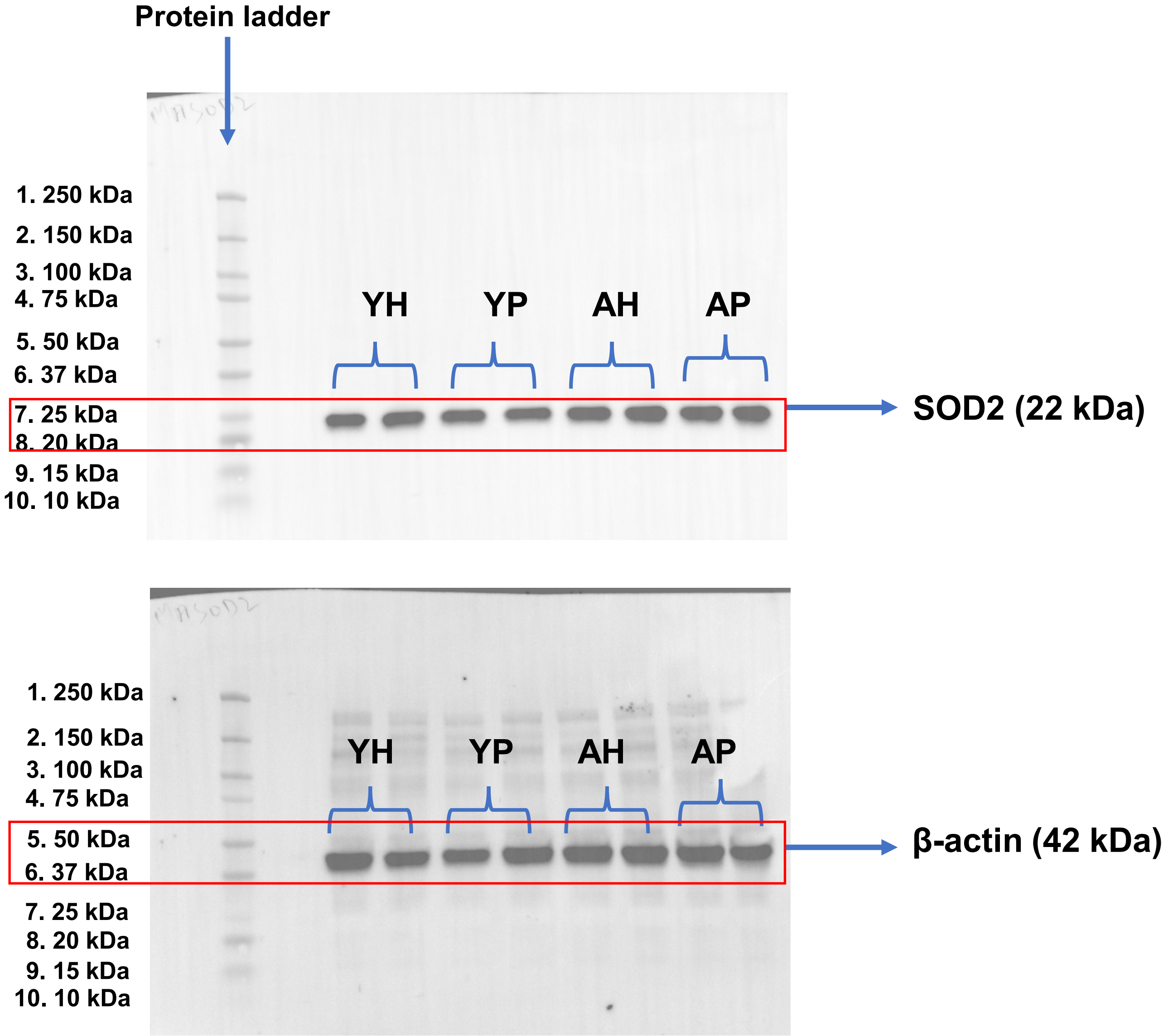

YH= Young Healthy
YP= Young Prediabetic
AH= Aged Healthy
AP= Aged Prediabetic

Blot-4 (8-28-2022)

Bio-rad ChemiDoc XRS+ System

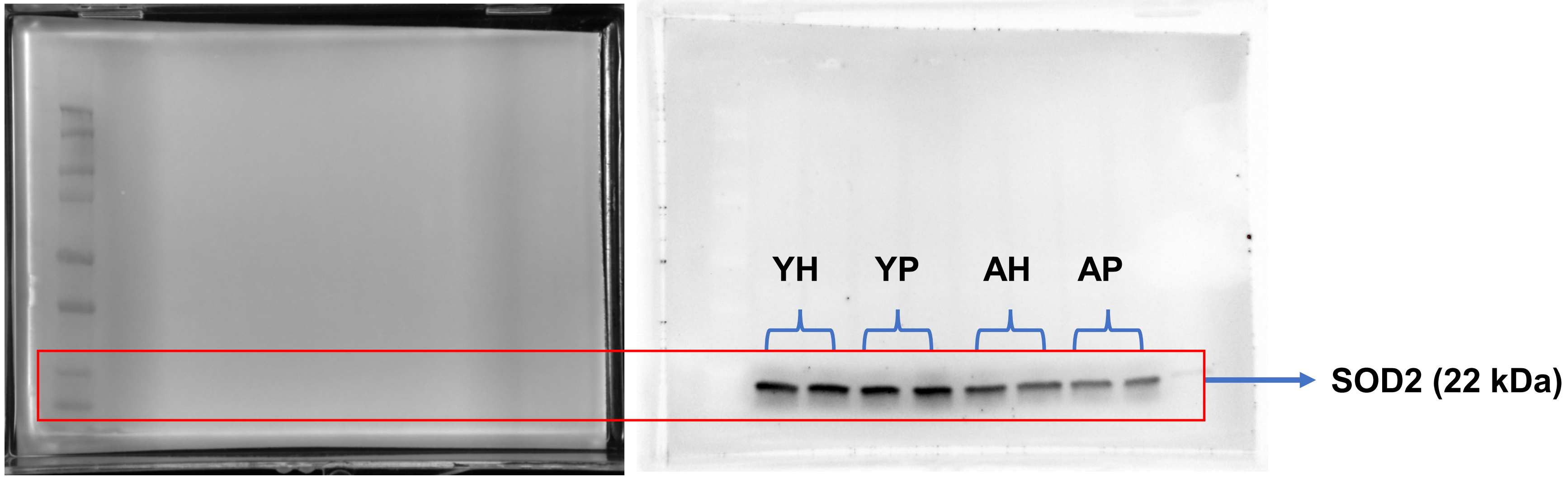

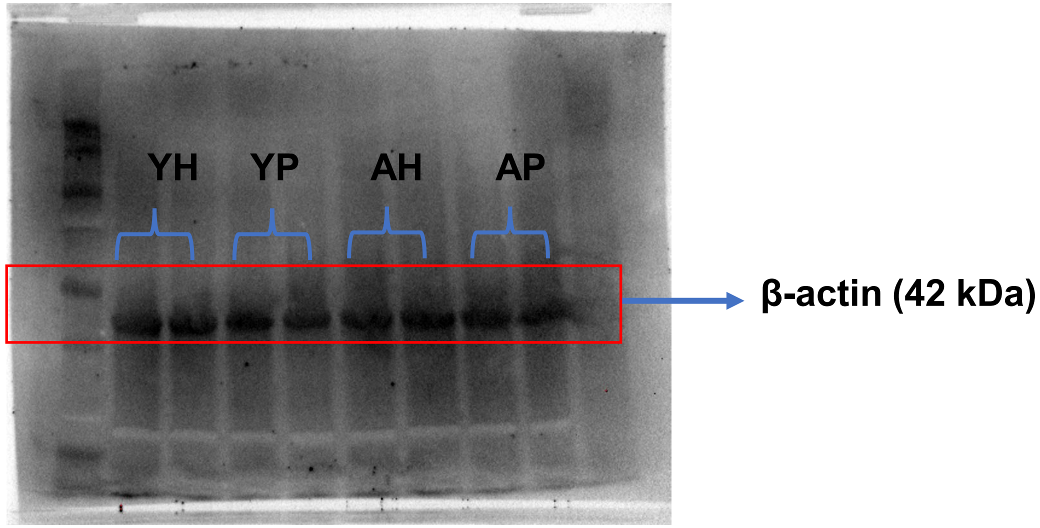

Blot-3 (8-14-2022)
Bio-rad ChemiDoc XRS+ System

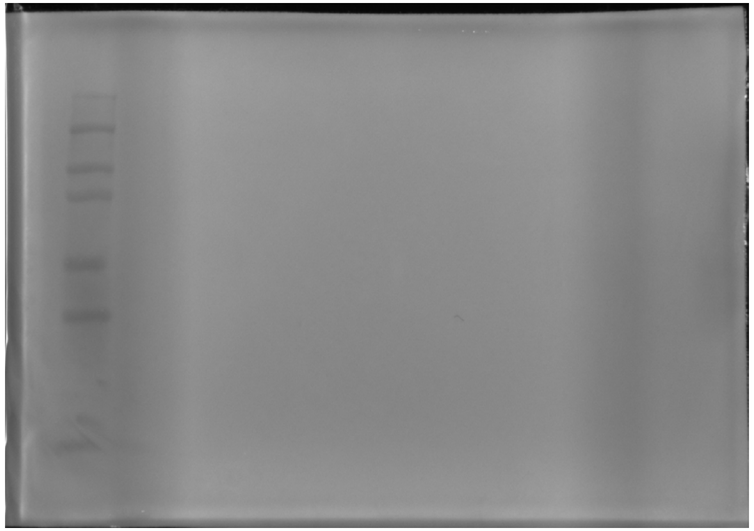

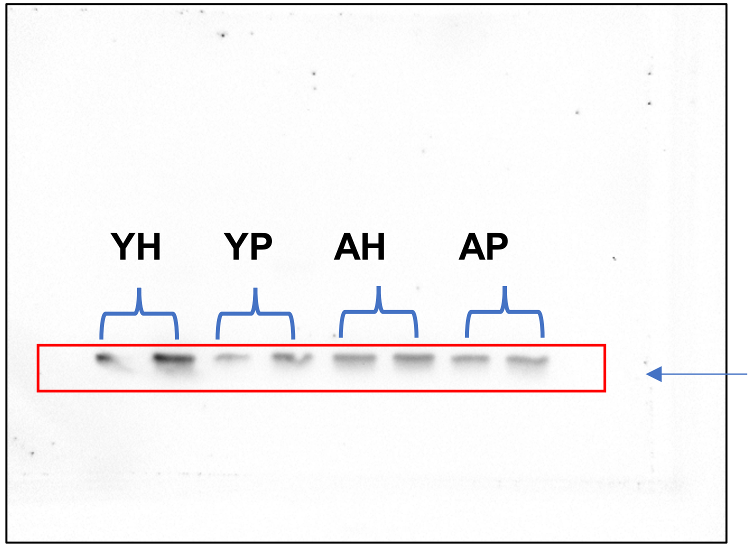

YH= Young Healthy
YP= Young Prediabetic
AH= Aged Healthy
AP= Aged Prediabetic

SOD2 (22 kDa)

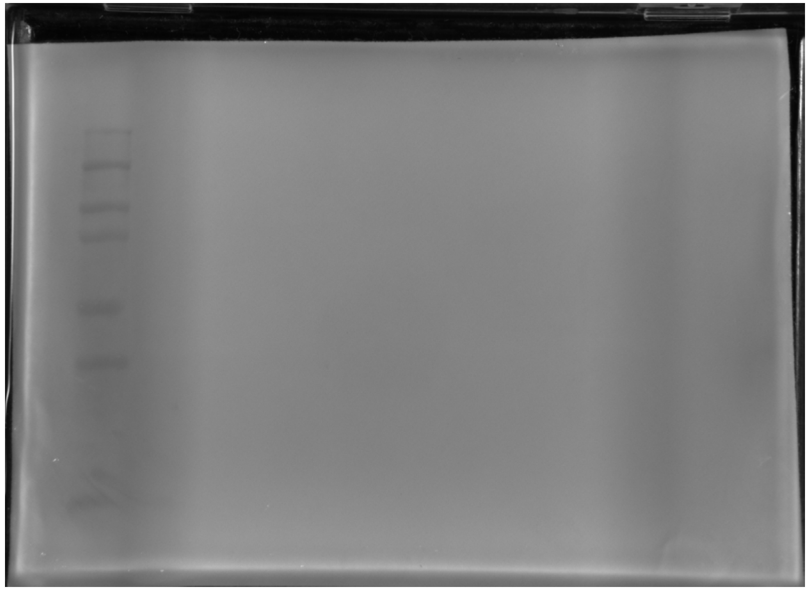

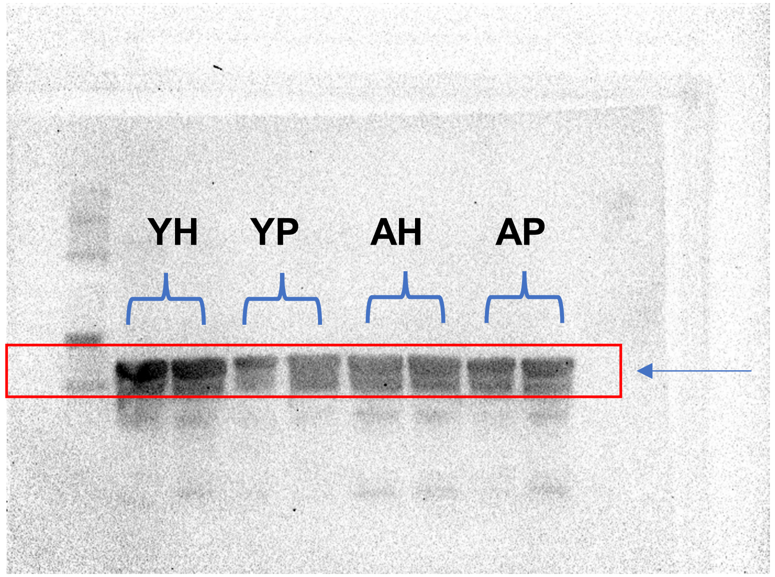

β-actin (42 kDa)

Blot-2 (7-30-2022)

Bio-rad ChemiDoc XRS+ System

YH= Young Healthy

YP= Young Prediabetic

AH= Aged Healthy

AP= Aged Prediabetic

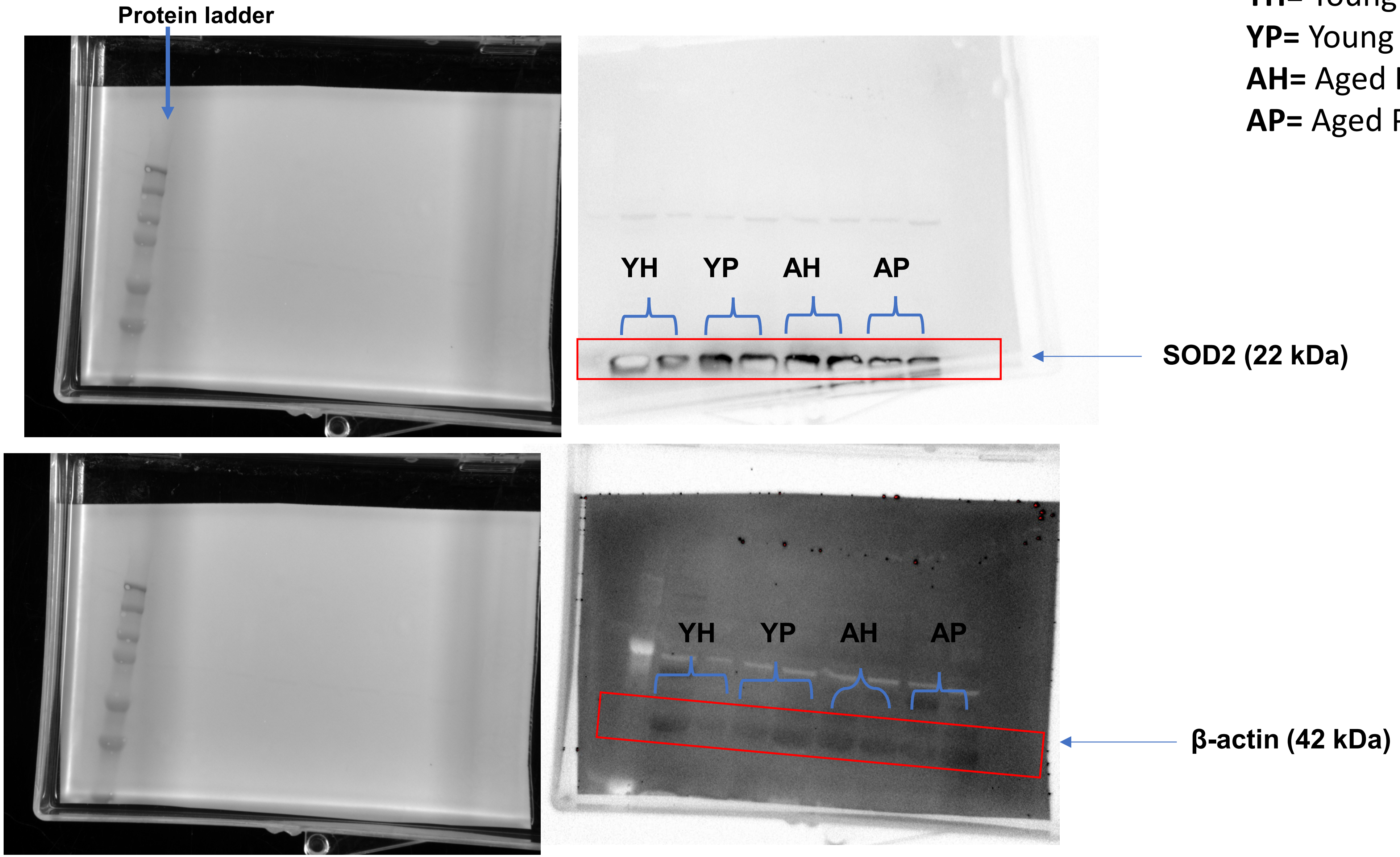

Blot-1 (6-29-2022)

Bio-rad ChemiDoc XRS+System

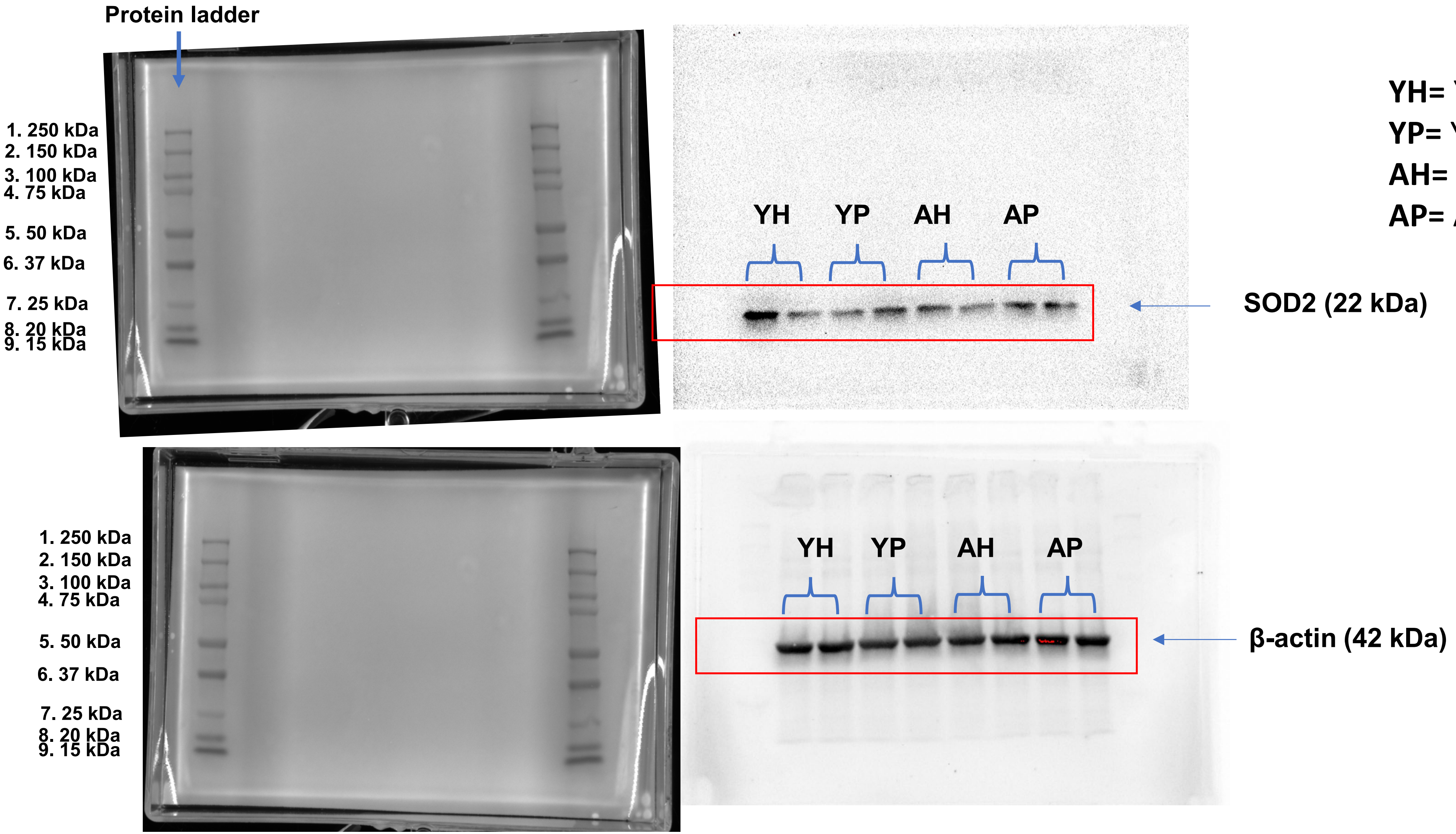

YH= Young Healthy
YP= Young Prediabetic
AH= Aged Healthy
AP= Aged Prediabetic
